# ZDHHC9 palmitoylates LAMTOR1 to promote renal cell carcinoma malignant progression

**DOI:** 10.1038/s41419-026-08558-8

**Published:** 2026-03-19

**Authors:** Bo Liu, Tao Hou, Xizhi Liu, Lu Liu, Zhiqiang Ma, Yujiao Zhang

**Affiliations:** 1 Department of Respiratory and Critical Care Medicine, The Second Affiliated Hospital of Xi’an Jiaotong University, Xi’an, China; 2 Department of Urology, The First Affiliated Hospital of Xi’an Jiaotong University, Xi’an, China; 3 Department of Pathology, Beth Israel Deaconess Medical Center, Harvard Medical School, Boston, MA USA; 4 Department of Respiratory and Critical Care Medicine, Xi’an Aerospace General Hospital, Xi’an, China; 5 Department of Medical Oncology, Senior Department of Oncology, Chinese PLA General Hospital, The Fifth Medical Center, Beijing, China

**Keywords:** Renal cell carcinoma, Post-translational modifications, Growth factor signalling

## Abstract

The lysosomal regulator complex member LAMTOR1 serves as a crucial pivot that recruits the mechanistic target of rapamycin complex 1 (mTORC1) to the lysosomal surface, thereby influencing biological processes such as cell growth and cancer progression. In renal cell carcinoma (RCC), existing studies reveal that mTORC1 signaling contributes to cancer progression. However, the precise regulatory mechanisms underlying mTOR signaling in RCC remain unclear and warrant further investigation. Here, we demonstrate that the palmitoylation enzyme Zinc Finger DHHC-Type Containing 9 (ZDHHC9) activates the mTOR signaling pathway, thereby accelerating cancer progression and highlighting its potential role in RCC. In our study, we identified that ZDHHC9 specifically palmitoylates LAMTOR1 at its Cys3/4 residues, enhancing the recruitment of mTORC1 and subsequently activating the mTOR signaling cascade. Collectively, our findings provide novel insights into the pathogenesis of RCC and establish ZDHHC9 as a key mediator of RCC progression through the palmitoylation of LAMTOR1, which may serve as a promising target for the diagnosis and treatment of this malignancy.

## Introduction

Renal cell carcinoma (RCC), which originates from the tubular epithelium, accounts for over 80% of renal malignancies and ranks among the top eight most common cancers, with an annual incidence increase of 1.6% [1]. Characterized by high invasiveness, strong metastatic potential, elevated mortality rates, and heterogeneous clinical progression, clear cell renal cell carcinoma (ccRCC) is associated with a poor prognosis [2, 3]. Surgical resection remains the primary treatment for localized tumors; however, approximately 25% of patients are diagnosed with advanced or metastatic disease at presentation [4]. Metastatic transformation is closely associated with poor clinical outcomes, as mRCC demonstrates resistance to conventional radiotherapy and chemotherapy, develops multidrug tolerance, and consequently leads to a less than 5-year survival rate for the majority of patients [5]. These factors highlight the pressing requirement for innovative therapeutic approaches to address this notoriously treatment-resistant malignancy.

Palmitoylation of proteins is a post-translational modification wherein fatty acyl moieties, typically 16-carbon palmitate, are covalently attached to cysteine residues of substrate proteins via the ZDHHC enzyme family. This process primarily regulates the trafficking and localization of substrate proteins among cell membranes, organelles, and nuclei [6]. Palmitoylation has been demonstrated to play a critical role in promoting liver cancer metastasis by modulating the CKAP4/EGFR signaling pathway and activating immunosuppressive cells, such as myeloid-derived suppressor cells [7–9]. In addition, approximately 150 cancer studies have identified palmitoylation enzymes ZDHHCx as potential contributors to the malignant progression of carcinomas [10, 11]. Meanwhile, it has been demonstrated that targeting ZDHHCx using their inhibitors, such as Cerulean or Orlistat, can enhance sensitivity to immune checkpoint therapy [12, 13]. Database analysis showed that the mRNA levels of ZDHHC9 in renal cell carcinoma (RCC) surpass those of other ZDHHCx family members and are significantly higher than in normal renal tissues [14]. The data from the cBioPortal database indicate that the amplification frequency of ZDHHC9 in renal cancer was approximately 1% to 2%. The *hTFtarget* database showed MYC, ERG, and CDK9 were potential transcription factors for ZDHHC9. Meanwhile, the *UALCAN* and *GEPIA 2* databases indicated that the mRNA levels of MYC, ERG, and CDK9 in renal cancer tissues are higher than those in normal renal tissues. In addition, MYC, ERG, CDK9, and ZDHHC9 are positively correlated in renal cancer. In bladder cancer, ZDHHC9 promotes tumor proliferation by mediating the palmitoylation of the binding immunoglobulin protein (BIP) at the Cys420 site, thereby inhibiting the unfolded protein response [15]. Furthermore, ZDHHC9 enhances PD-L1 expression on T cells via the IFN-γ-induced JAK-STAT1 signaling pathway, leading to reduced cytotoxicity of T cells [16]. Additionally, a mouse pain model confirmed that ZDHHC9 increases morphine tolerance through palmitoylation of apelin receptors [17]. while clinical research indicates that ZDHHC9 levels are positively correlated with neuropathic cancer pain [18]. Thus, elucidating the molecular mechanism of ZDHHC9 in RCC could provide a novel and comprehensive strategy for targeted therapy.

It is well-established that the mechanistic target of rapamycin (mTOR) plays a critical role in cellular processes, and its dysregulation contributes to tumor development and angiogenesis [19]. More than half of renal cell carcinoma (RCC) clinical specimens exhibit high expression levels of phosphorylated mTOR and its downstream effector, Ribosomal Protein S6 [20]. Despite the use of rapamycin (an mTOR inhibitor) as a therapeutic strategy for RCC, some patients develop drug resistance, leading to suboptimal responses [21]. LAMTOR1, as a component of the lysosomal regulatory complex, facilitates the recruitment of mTORC1 to the lysosomal surface, thereby activating the mTOR signaling pathway [22]. It has been reported that the palmitoylation of LAMTOR1 significantly increases prior to mTORC1 activation [23]. Consequently, targeting the palmitoylation enzyme of LAMTOR1 and inhibiting its palmitoylation in combination with other therapies may represent a promising novel strategy for RCC treatment.

In this study, we confirmed that ZDHHC9 promotes the malignant progression of renal cell carcinoma (RCC). Moreover, the combination of ZDHHC9 knockdown and rapamycin treatment achieved a more pronounced tumor inhibition effect. Further mechanistic investigation revealed that ZDHHC9 mediates the palmitoylation of LAMTOR1 at its Cys3/4 sites, thereby recruiting mTOR to the lysosomal surface and promoting RCC proliferation. These findings suggest that ZDHHC9 may serve as a potential therapeutic target for RCC.

## Materials and methods

### Cell lines and culture

Mouse renal cancer cell line Renca, human RCC cell lines 769-P, SW839, 786-O, and HEK-293T cells were purchased from the American Type Culture Collection and cultured in RPMI-1640 medium or DMEM (Gibco; Thermo Fisher Scientific, Inc.) supplemented with 10% fetal bovine serum (FBS) (Gibco; Thermo Fisher Scientific, Inc.), 100 U/ml penicillin and 0.1 mg/ml streptomycin (Gibco; Thermo Fisher Scientific, Inc.) at a temperature of 37 °C.

### Reagents and antibodies

Rapamycin (cat. no. HY-10219), AZD-8055 (cat. no. HY-10422), 2-Bromopalmitate (cat. no. HY-111770), and Methylthiazolyldiphenyl-tetrazolium (cat. no. HY-15924) were purchased from MedChemExpress. Primary antibodies against ZDHHC9 (cat. no. PA2657; 1:1000), LAMTOR1(cat. no. PS06321; 1:1000), Phospho-p70 S6 Kinase (cat. no. TA3228M; 1:1000), p70 S6 Kinase Antibody (cat. no. T55365S; 1:1000), Ribosomal Protein S6 (cat. no. PU741963S; 1:1000), Phospho-S6 Ribosomal Protein (cat. no. TP56465M; 1:1000), Bax (cat. no. TA0120S; 1:1000), Cleaved PARP (cat. no. T55035S; 1:1000), Cleaved-Caspase 3 (cat. no. TA7022S; 1:1000), Vinculin (cat. no. T55164S; 1:1000), Ki67 (cat. no. TW0001S; 1:1000), Phospho-mTOR (cat. no. PC3495S; 1:1000), mTOR (cat. no. T55306S; 1:1000), HA-Tag (cat. no.TT0050S; 1:1000), DYKDDDDK-Tag/FLAG (cat. no. M20008M; 1:1000), GST-Tag (cat. no. M20007M; 1:1000) were purchased from Abmart. ZDHHC9 (cat. no. 24046-1-AP; 1:100) and Phospho-S6 Ribosomal Protein (cat. no. 67898-1-Ig; 1:100) were purchased from Proteintech. Secondary Goat Anti-Rabbit IgG (cat. no. ab205718; 1:2000) and Goat Anti-Mouse IgG (cat. no. ab150113; 1:2000) were purchased from Abcam.

### Plasmids

HA-ZDHHC9, Flag-LAMTOR1, GST-LAMTOR1, Flag-mTOR, and Flag-TMEM192 were purchased from MIAOLING PLASMID. HA-tagged ZDHHC9-truncated, HA-ZDHHC9^*C169S*^, Flag-LAMTOR1^*C3S*^, Flag-LAMTOR1^*C4S*^, Flag-LAMTOR1^*2CS*^, and GST-LAMTOR1^*2CS*^ were constructed according to KOD-Plus-Mutagenesis Kit (cat. no. SMK-101; TOYOBO). All plasmid constructs were verified by DNA sequencing.

### MTT assay

769-P and SW839 were plated into 96-well plates at a density of 3.0 × 10^4^ cells/ml and treated with different processing conditions for 0, 24, 48, 72, 96 h. Subsequently, 180 μl 10% MTT solution (dissolved in serum-free RPMI-1640 medium; 1:9) was added into each well and incubated for 3 h. Then the supernatant was abandoned, then 180 μl DMSO was added to each well to dissolve the formazan crystals by oscillating it on a mini shaker for 10 min. The absorbance was measured at 490 nm using a microplate reader (Bio-Rad Laboratories Inc.).

### Western blotting

Washed with phosphate-buffered saline (PBS), cells or ground tissues were mixed with cell lysates containing protease inhibitors and phosphatase inhibitors, centrifuged at 13,000 × *g* for 12 min. Protein concentration was detected via the BCA kit. 4 × protein loading buffer was added and heated at 100 °C for 10 min to denature the proteins. A total of 30 µg of protein samples were added to 8%, 10%, or 15% SDS-PAGE, electrophoresed, and transferred to polyvinylidene fluoride (PVDF) membrane. PVDF membrane was blocked in milk at room temperature for 1 h, incubated with primary antibody at 4 °C overnight, and then incubated with peroxidase-conjugated secondary antibody at room temperature for 1 h (RCA, USA) and analyzed with Image Lab 5.1.

### Co-immunoprecipitation

After transfection with the specific plasmid, the HEK-293T cells were lysed with IP buffer [50 mM Tris HCl, 150 mM NaCl, 1 mM ethylenediaminetetraacetic acid (EDTA), 1% Triton X-100] containing protease inhibitors (Sigma-Aldrich; Merck KGaA) and phosphatase inhibitors (Sigma-Aldrich; Merck KGaA). After incubating with M2 agarose beads (Sigma-Aldrich) and with anti-FLAG, anti-HA, or anti-GST antibodies for 3 h at 4 °C with gentle shaking (15 r/min), the cell lysate was washed with IP buffer three times, and the target protein was extracted from the beads by boiling at 100 °C for 10 min.

### Immunohistochemical assay

The renal cancer tissues microarrays were deparaffinized, rehydrated, endogenous peroxidase blockaded and antigen repaired orderly. Subsequently, tumor sections were blocked with 5% BSA for 30 min and incubated overnight with primary ZDHHC9 (cat. no. PA2657; 1:200). Incubated with secondary antibodies for 1 h, tissue microarrays were visualized using the DAB reagent according to the manufacturer’s instructions. Three fields (×100) were randomly selected from each section for analysis.

### Immunofluorescence

After treatment with 1 µM LysoSensor™ Probes (cat. no. A66463; Thermo Fisher) for 1 h, 769-P or SW839 cells were fixed with 4% paraformaldehyde for 30 min at room temperature. The cells were then incubated with primary mTOR (cat. no. T55306S; 1:100) overnight at 4 °C. After incubation with the primary antibody, cells were incubated with Cy3-labeled goat anti-rabbit IgG secondary antibody (Beyotime, Biotechnology; cat. no. A0516) for 1 h at room temperature, and counterstained with DAPI-Aqueous, Fluoroshield (Abcam; cat. no.104139) at room temperature for 20 min. Target protein distribution was captured using a fluorescence microscope.

### Acyl-biotin exchange assay

HEK-293T cells overexpressing HA-tagged ZDHHC9 and FLAG-tagged LAMTOR1 were obtained 48 h after transfection. The transfected HEK293T cells were lysed with IP buffer containing protease inhibitors (Sigma-Aldrich; Merck KGaA), phosphatase inhibitors (Sigma-Aldrich; Merck KGaA), and N-ethylmaleimide (NEM) (Aladdin; cat. no. E100553) dissolved in 100% EtOH. Cells were then suspended for 2 h at room temperature. And the protein in the suspension was precipitated with acetone, IP buffer with hydroxylamine (Aladdin; cat. no. H112477) was added, and then rotated at room temperature for 2 h after ultrasound. And the protein in the suspension was precipitated with acetone again, the precipitate was incubated with 20 μM Biotin-BMCC in IP Buffer at 4 °C for 2 h. Then 40 µl streptavidin Agarose (Beyotime; cat. no. P-2159) was added to the suspension for rotating at 4 °C for 2 h. The beads were washed with IP Buffer for 3 times and boiled for 10 min at 100 °C.

### Flow cytometry assay

Annexin V-FITC/7-AAD kit (cat. no. 40311ES60; Yeasen) was added to 100 µl prepared cell suspensions for 20 min at room temperature. After centrifugation, the supernatant was discarded. After washing and centrifugation with 150 µl PBS three times, the supernatant was discarded and analyzed according to flow cytometry (BD FACSCanto flow cytometer, BD Biosciences).

### Xenograft animal model

All animal care and experiments were authorized by the Institutional Animal Care and Use Committee of Xi’an Jiaotong University. And the Animal Ethics Committee approval number is No. 2021.904. The health and behavior of the mice were monitored daily. Based on the relevant literature in this field [24–26]. and the 3 R principles, we selected 5 mice of the same strain, of the same gender, of the same age, and with the same weight for each group, all from the inbred strain. SW839 cells were resuspended in serum-free RPMI-1640 medium at a density of 1.0 × 10^7^ cells/ml. Subsequently, 4.0 × 10^6^ cells in suspension solution were subcutaneously injected into the right flank of BALB/c Nude mice (age, 4 weeks), and upon the tumor volume reaching ~50 mm^3^ in size, the mice were numbered using an ear marker, and RandoMice software was used to randomize the mice according to assay design. The tumor volume was calculated using the following formula: volume (mm^3^) = 0.5 × length × width^2^. When the tumor volume exceeded 1000 mm^3^, mice were sacrificed by CO_2_ (30% of the chamber volume/min) and tumors were harvested; the animals were exposed to CO_2_ until complete cessation of breathing was observed for 15 min. The investigator was blinded to the group allocation during the experiment and assessed the outcome. During the measurement of the tumor length and width, as well as its weighing process, the recorders were unaware of the specific group to which the animals belonged.

### Ethics statement

All experimental methods were strictly performed in accordance with the relevant guidelines and regulatory requirements of the Ethics Committee of Xi’an Jiaotong University. The clinical samples used were obtained with the informed consent of the donors at the time of collection and were authorized by the Ethics Committee of Xi’an Jiaotong University.

### Statistical analysis

All the data were presented as mean ± SD of three independent experiments. All statistical analyses were performed using GraphPad Prism 8.0.2 software (GraphPad Software, Inc.). A Student’s *t* test was used for the comparisons between two groups and *p* < 0.05 was considered a statistically significant difference.

## Result

### ZDHHC9 is overexpressed and acts as an oncogene in RCC

**Fig. 1:**
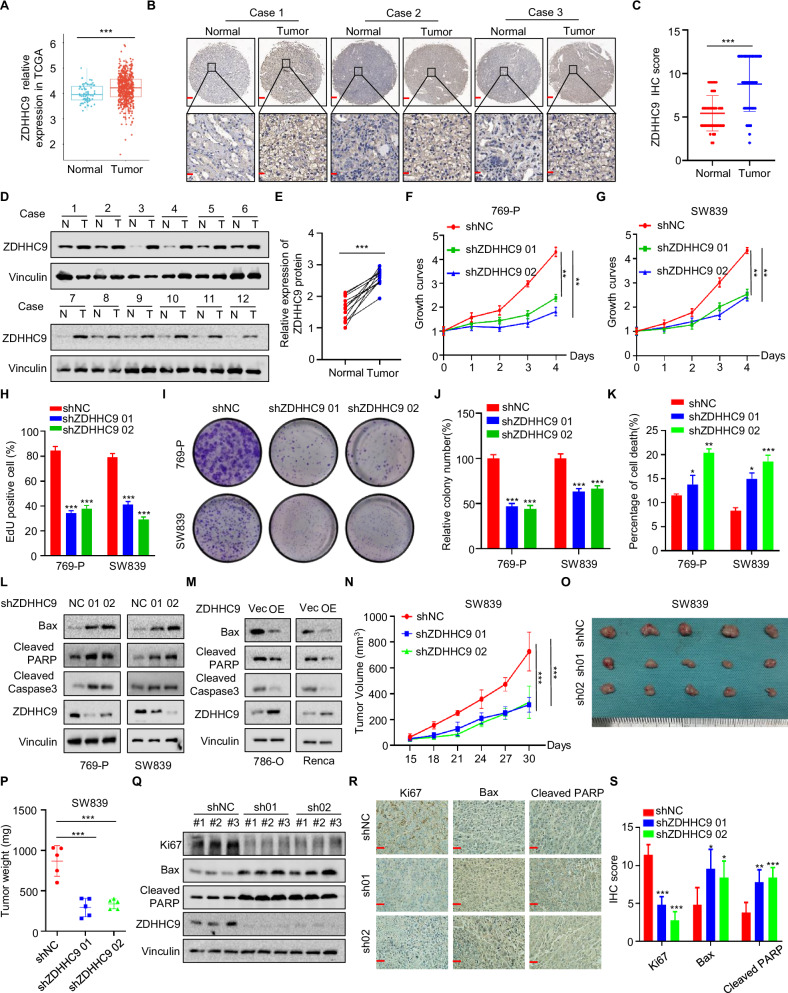
ZDHHC9 is overexpressed and acts as an oncogene in RCC. **A** The ZDHHC9 expression of normal and RCC samples in the TCGA database. ***p < 0.001. **B** Representative images of ZDHHC9 immunohistochemistry staining in normal tissue and RCC tissue samples. Scale bars, upper 500 μm, lower 100 μm. **C** Statistical analysis of ZDHHC9 expression in normal tissue and RCC tissue samples. ***p < 0.001. **D** Western blotting analysis of ZDHHC9 protein expression in normal and RCC tissues from patients. **E** The quantitative analysis of the relative expression of ZDHHC9 protein. ***p < 0.001. **F**, **G** MTT assay was performed to detect the cell viability in 769-P and SW839 with ZDHHC9 knockdown. **p < 0.01. **H** Statistic analysis of EdU assay that was performed to detect proliferation in 769-P and SW839 cells with ZDHHC9 knockdown. ***p < 0.001. **I**, **J** Colony formation assays and quantification results of 769-P and SW839 cells treated with ZDHHC9 knockdown. ***p < 0.001. **K** Statistic analysis of flow cytometry analysis for detecting apoptotic cells in 769-P and SW839 cells with ZDHHC9 knockdown. *p < 0.05, **p < 0.01, ***p < 0.001. **L**, **M** Western blotting was performed to detect the effect of ZDHHC9 on the expression of apoptosis markers (Bax, Cleaved PARP, Cleaved Caspase3) in 769-P, SW839, 786-O, and Renca cells. **N** The growth curves of xenografts in different groups. Tumor volumes were measured every 3 days from day 15 to day 30. ***p < 0.001. After 30 days, the nude mice were sacrificed, **O** illustration of tumors excised from male nude mice in each group, and **P** SW839 xenografts were weighed. ***p < 0.001. **Q** Western blotting analysis of Ki67, Bax, Cleaved PARP, and ZDHHC9 expression in tumor xenografts. **R**, **S** Immunohistochemical analysis and quantification results of Ki67, Bax, and Cleaved PARP in tumor xenografts. (Scale bar, 200 µm) *p < 0.05, **p < 0.01, ***p < 0.001. All data are presented as mean ± SD of three independent experiments.

To confirm the role of ZDHHC9 in RCC, we first analyzed the expression levels of ZDHHC9 in RCC and normal tissue samples via the TCGA database. The results demonstrated that ZDHHC9 expression was significantly elevated in RCC tissues compared to normal tissues (Fig. 1A). Additionally, immunohistochemistry and western blotting analyses of tissue samples from RCC patients revealed markedly higher ZDHHC9 expression levels in RCC tissues than in normal tissues (Fig. 1B–E). To select appropriate cell lines, we examined the expression levels of ZDHHC9 in various RCC cell lines (Fig. S1A, B) and subsequently chose 769-P and SW839 for constructing ZDHHC9 knockdown cell lines, as well as 786-O and Renca for establishing overexpression cell lines. The MTT assay conducted in 769-P and SW839 cells confirmed that, compared with the control group, the cell proliferation capacity was significantly impaired in the ZDHHC9 knockdown group (Fig. 1F, G). On the contrary, the MTT experiment performed on 786-O and Renca cells demonstrated that, compared with the control group, cell proliferation capacity was significantly increased in the ZDHHC9 overexpression group (Fig. S1C, D). Consistently, further EdU and plate cloning assays yielded results analogous to those observed in the MTT experiment (Figs. 1H–J and S1G–I). To investigate whether ZDHHC9 influences the proliferative capacity of RCC cells via apoptosis, flow cytometry analysis demonstrated that ZDHHC9 knockdown significantly promoted cellular apoptosis, whereas ZDHHC9 overexpression markedly reduced apoptosis (Figs. 1K and S1J, K). Consistently, western blotting results further corroborated these findings by analyzing the key apoptosis-related proteins, including Bax, Cleaved PARP, and Cleaved Caspase3 (Fig. 1L, M). As illustrated in Fig. 1N–P, ZDHHC9 knockdown effectively inhibited xenograft tumor growth and reduced tumor weight in vivo. Additionally, immunohistochemistry and western blotting analyses confirmed that ZDHHC9 knockdown decreased the expression of Ki67, a proliferation marker, while increasing the expression of pro-apoptotic proteins Cleaved Caspase3, Bax, and Cleaved PARP (Fig. 1Q–S). Collectively, these results indicate that ZDHHC9 plays a critical role in promoting tumor proliferation in RCC.

### ZDHHC9 activates the mTOR signaling pathway in RCC

**Fig. 2:**
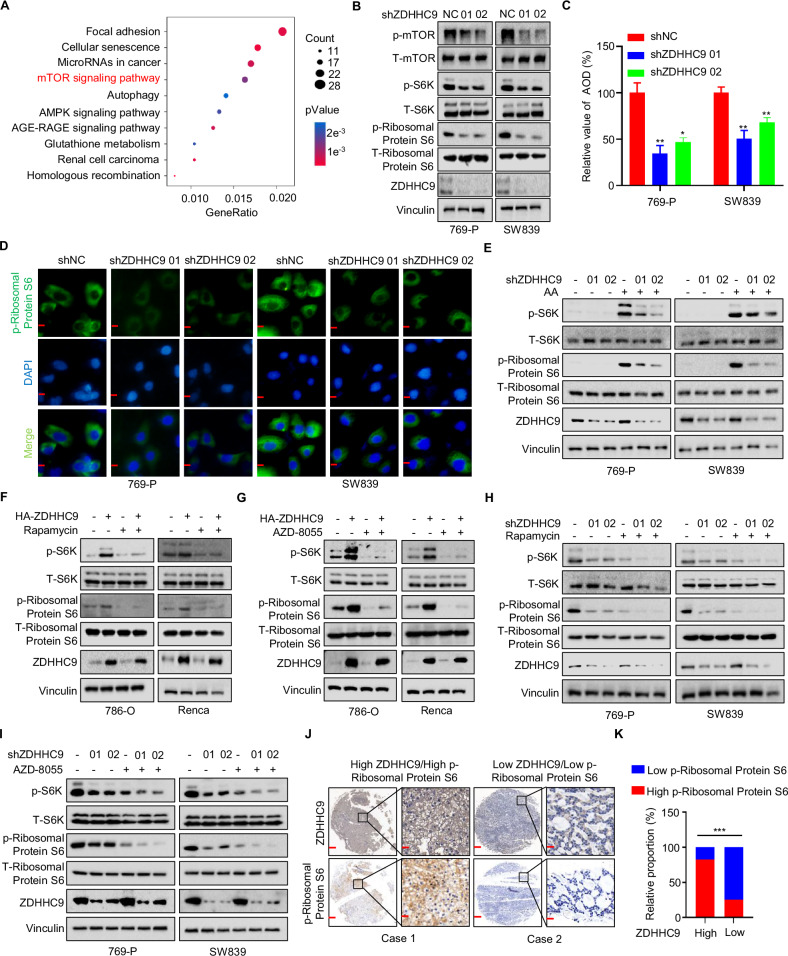
ZDHHC9 activates the mTOR signaling pathway in RCC. **A** RNA sequencing GO enrichment results suggested that ZDHHC9 knockdown affected mTOR signaling pathway. **B** Western blotting assay of p-mTOR, T-mTOR, p-S6, T-S6, p-Ribosomal Protein S6, T-Ribosomal Protein S6 and ZDHHC9 in 769-P and SW839 cells with ZDHHC9 knockdown. **C** Statistic analysis of immunofluorescence that performed to detect expression of p-Ribosomal Protein S6 in 769-P and SW839 cells with ZDHHC9 knockdown. *p < 0.05. **p < 0.01. **D** Immunofluorescence assays that performed to detect expression of p-Ribosomal Protein S6 in 769-P and SW839 cells with ZDHHC9 knockdown. (Scale bar, 200 µm). **E** 769-P and SW839 shNC**/**shZDHHC9 cells were subjected to amino acid starvation for 6 h and re-supplementation for 1 h. p-S6, T-S6, p-Ribosomal Protein S6, T-Ribosomal Protein S6 and ZDHHC9 were detected. 786-O and Renca Vec/OE ZDHHC9 cells were treated with or without **F** Rapamycin (20 μM; 24 h) / **G** AZD-8055 (50 nM; 24 h). Markers in **E** were detected by western blotting. 769-P and SW839 shNC/shZDHHC9 cells treated with or without **H** Rapamycin (20 μM; 24 h) / **I** AZD-8055 (50 nM; 24 h). Markers in **E** were detected by western blotting. **J** The ZDHHC9 and p-Ribosomal Protein S6 expression differed between different RCC tissue microarray. Scale bars, red 500 μm, black 100 μm. **K** Statistic analysis of (**J**). ***p < 0.001. All data are presented as mean ± SD of three independent experiments.

Our previous results demonstrated that ZDHHC9 promotes the proliferation of RCC cells. However, the underlying mechanism remains unclear. To investigate how ZDHHC9 influences tumor malignancy, we performed RNA-seq analysis to identify potential pathways regulated by ZDHHC9. Both GO enrichment analysis (Fig. 2A) and KEGG enrichment analysis (Fig. S2A) revealed that ZDHHC9 knockdown affects the mTOR signaling pathway. Additionally, RPS6KA5, which encodes ribosomal protein S6, a downstream molecule of mTOR, ranked among the top ten downregulated genes upon ZDHHC9 knockdown (Fig. S2B). Subsequently, western blotting was employed to confirm whether ZDHHC9 regulates the mTOR pathway. Compared with the control group, the expression levels of key mTOR pathway indicators (p-mTOR, p-Ribosomal Protein S6, and p-S6K) were significantly reduced in the ZDHHC9 knockdown group (Fig. 2B) and enhanced in the ZDHHC9 overexpression group (Fig. S2C). Further immunofluorescence assays corroborated the western blotting results (Figs. 2C, D and S2D, E). Additionally, we subjected 769-P and SW839 cells to amino acid (AA) starvation, followed by replenishment of AAs. Western blotting analysis revealed that, compared with the control group, the increase in p-S6K and p-Ribosomal Protein S6 expression levels was abolished in ZDHHC9 knockdown groups, indicating that ZDHHC9 knockdown inhibits the recovery of the mTOR pathway (Fig. 2E). Conversely, similar experiments conducted in 786-O and Renca cells demonstrated that ZDHHC9 overexpression promotes the recovery of the mTOR pathway (Fig. S2F). Rapamycin and AZD-8055 are well-established mTOR inhibitors, and multiple studies have confirmed their ability to downregulate the mTOR pathway [27–30]. Western blotting was employed to assess the expression levels of p-S6K and p-Ribosomal Protein S6 following treatment with Rapamycin or AZD-8055. In 786-O and Renca cells, both Rapamycin and AZD-8055 effectively suppressed mTOR pathway activation induced by ZDHHC9 overexpression (Fig. 2F, G). Furthermore, in 769-P and SW839 cells, these treatments inhibited mTOR signaling, with a more pronounced effect observed in ZDHHC9 knockdown cells (Fig. 2H, I). Moreover, our findings revealed a positive correlation between ZDHHC9 expression levels and p-Ribosomal Protein S6 in RCC specimens (Fig. 2J, K). Collectively, these results suggest that ZDHHC9 upregulates the mTOR signaling pathway in RCC.

### ZDHHC9 interacts with LAMTOR1

**Fig. 3:**
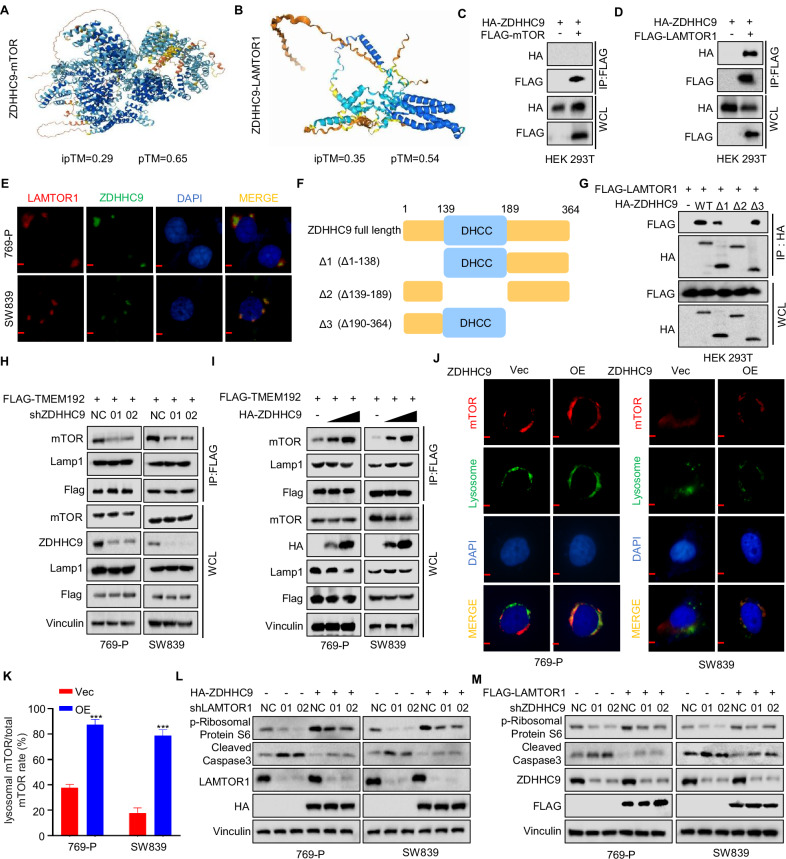
ZDHHC9 interacts with LAMTOR1. **A**, **B** AlphaFold3 was used to predict the possibility of ZDHHC9 binding to mTOR and LAMTOR1. **C** HEK-293T cells were transfected with FLAG-mTOR and HA-ZDHHC9. The cell lysates were subjected to immunoprecipitation with anti-FLAG. **D** HEK-293T cells were transfected with FLAG-LAMTOR1 and HA-ZDHHC9. The cell lysates were subjected to immunoprecipitation with anti-FLAG. **E** Confocal immunofluorescence microscopic analysis of ZDHHC9 and LAMTOR1 in 769-P and SW839 cells. (Scale bar, 50 µm). **F** Overview of ZDHHC9 structures. **G** HEK-293T cells transfected with the indicated constructs were subjected to immunoprecipitation with anti-FLAG or anti-HA. **H** 769-P and SW839 shNC/shZDHHC9 cells transfected with the FLAG-TMEM192 were subjected to immunoprecipitation with anti-FLAG. **I** 769-P and SW839 cells transfected with the FLAG-TMEM192 and gradient HA-ZDHHC9 were subjected to immunoprecipitation with anti-FLAG. **J**, **K** Confocal immunofluorescence microscopic analysis of mTOR and lysosome in 769-P and SW839 Vec/OE ZDHHC9 cells. (Scale bar, 50 µm). ***p < 0.001. **L** 769-P and SW839 shNC/shZDHHC9 cells transfected with or without FLAG-LAMTOR1 were subjected to detect the level of p-Ribosomal Protein S6 and Cleaved Caspase3. **M** 769-P and SW839 shNC/shLAMTOR1 cells transfected with or without HA-ZDHHC9 were subjected to detect the level of p-Ribosomal Protein S6 and Cleaved Caspase3. All data are presented as mean ± SD of three independent experiments.

Previous studies have demonstrated that mTOR and LAMTOR1 undergo palmitoylation, which stimulates downstream molecules in the mTOR signaling pathway [23]. To investigate whether ZDHHC9 is involved in this process, we predicted the potential binding of ZDHHC9 to mTOR and LAMTOR1. The results indicated that both LAMTOR1 and mTOR are likely to interact with ZDHHC9 (Fig. 3A, B). The binding energy prediction of AlphaFlod-Multimer and the MM/GBA analysis indicate that the binding of mTOR, LAMTOR1 and ZDHHC9 is stable (Fig. S3A). To validate these predictions, we co-transfected HEK-293T cells with HA-ZDHHC9 and Flag-mTOR or Flag-LAMTOR1. Immunoprecipitation (IP) analysis revealed that ZDHHC9 specifically co-immunoprecipitated with LAMTOR1 but not with mTOR (Fig. 3C, D). The same conclusion was also drawn from the endogenous IP results in 769-P and SW839 cells (Fig. S3B). The IP experiments of LAMTOR1 with the ZDHHCx family indicated that only ZDHHC9 could interact with LAMTOR1 (Fig. S3C). Furthermore, immunofluorescence assays confirmed the co-localization of ZDHHC9 and LAMTOR1 in 769-P and SW839 cells (Fig. 3E). To delineate the specific region responsible for this interaction, we generated three truncated mutants of ZDHHC9 tagged with an HA epitope (Fig. 3F) and co-transfected them with Flag-tagged LAMTOR1. IP experiments demonstrated that the Δ2 region (Δ139-189aa) of ZDHHC9 failed to physically interact with LAMTOR1 (Fig. 3G). LAMTOR1 activates the mTOR signaling pathway by recruiting mTORC1 to the lysosomal surface for activation by Rheb (Ras homolog enriched in brain, a GTPase) [31]. The tag plasmid designed for TMEM192, a protein involved in the functional characterization of lysosomal membrane proteins, was utilized to perform the lysosome isolation experiment [32, 33]. We separately transfected Flag-TMEM192 into 769-P and SW839 cells. The lysosome IP results demonstrated that, compared with the control group, ZDHHC9 knockdown cells exhibited reduced lysosomal mTOR levels, whereas ZDHHC9 overexpression cells showed increased lysosomal mTOR levels (Fig. 3H, I). Additionally, immunofluorescence assays revealed that ZDHHC9 overexpression promoted the translocation of mTOR to lysosomes in 769-P and SW839 cells (Fig. 3J, K). Furthermore, western blotting experiments confirmed that LAMTOR1 knockdown attenuated the ZDHHC9 overexpression-mediated increase in p-S6K and decrease in Cleaved Caspase3 (Fig. 3L). Similarly, ZDHHC9 knockdown diminished the effects induced by LAMTOR1 overexpression on these indices (Fig. 3M). These findings collectively suggest that ZDHHC9 interacts with LAMTOR1 and activates the mTOR pathway.

### ZDHHC9 palmitoylates LAMTOR1 to recruit mTOR

**Fig. 4:**
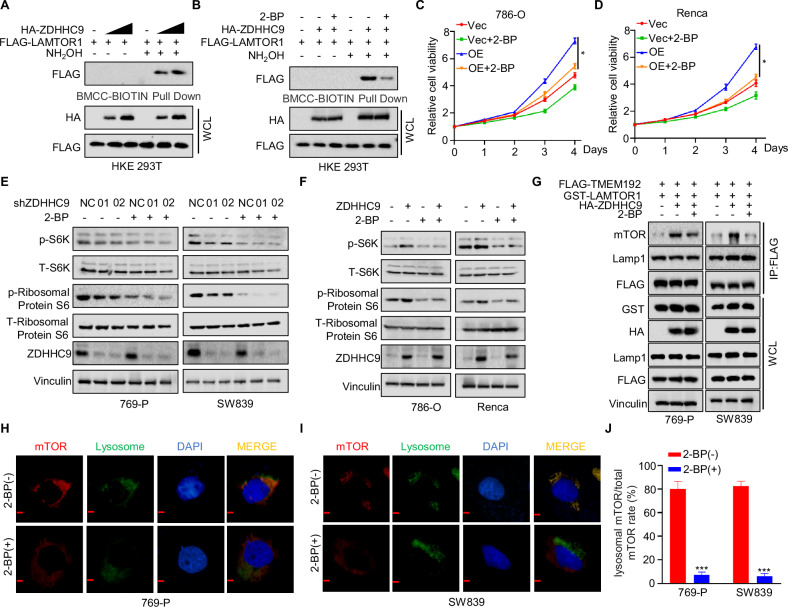
ZDHHC9 palmitoylates LAMTOR1 to recruit mTOR. **A** HEK-293T cells were transfected with FLAG-LAMTOR1 and gradient HA-ZDHHC9. The cell lysates were subjected to conduct to ABE assays. **B** HEK-293T cells were transfected with FLAG-LAMTOR1 and HA-ZDHHC9. After treated with or without 2-BP (50 μM) for 12 h, the cell lysates were subjected to conduct to ABE assays. **C**, **D** MTT assay was performed to detect the cell viability in 769-P and SW839 Vec/OE ZDHHC9 cells with or without 2-BP (50 μM) for 12 h. **E** 769-P and SW839 shNC/shZDHHC9 cells were subjected to 2-BP (50 μM) treatment for 12 h. p-S6, T-S6, p-Ribosomal Protein S6, T-Ribosomal Protein S6 and ZDHHC9 were detected. **F** 786-O and Renca Vec/OE ZDHHC9 cells were subjected to 2-BP (50 μM) treatment for 12 h. p-S6, T-S6, p-Ribosomal Protein S6, T-Ribosomal Protein S6 and ZDHHC9 were detected. **G** 769-P and SW839 cells transfected with the FLAG-TMEM192, HA-ZDHHC9 and GST-LAMTOR1. After treated with or without 2-BP (50 μM) for 12 h, the cell lysates were subjected to immunoprecipitation with anti-FLAG. **H**–**J** Confocal immunofluorescence microscopic analysis of mTOR and lysosome in 769-P and SW839 cells treated with or without 2-BP (50 μM) for 12 h. (Scale bar, 50 µm). ***p < 0.001. All data are presented as mean ± SD of three independent experiments.

In order to investigate whether LAMTOR1 undergoes ZDHHC9-mediated palmitoylation, we co-transfected HEK-293T cells with HA-ZDHHC9 and FLAG-LAMTOR1. The Acyl-Biotin Exchange (ABE) assay confirmed that ZDHHC9 enhances the palmitoylation level of LAMTOR1 in a concentration-dependent manner (Fig. 4A). Furthermore, additional ABE analysis demonstrated that 2-Bromohexadecanoic acid (2-BP), a specific palmitoylation inhibitor, significantly reduced the palmitoylation level of LAMTOR1 mediated by ZDHHC9 (Fig. 4B). As shown by MTT assays (Fig. 4C, D), 2-BP suppressed the cell proliferation induced by LAMTOR1 overexpression in RCC. Moreover, western blotting results revealed that the inhibitory effect of 2-BP on the mTOR pathway was more pronounced in ZDHHC9 knockdown cells in 769-P and SW839 cell lines (Fig. 4E) and effectively attenuated the mTOR pathway activation caused by ZDHHC9 overexpression (Fig. 4F). Subsequent lysosome immunoprecipitation (IP) assays indicated that the recruitment of mTOR to lysosomes by LAMTOR1 was also inhibited by 2-BP treatment (Fig. 4G). Consistently, immunofluorescence analysis showed that the proportion of mTOR localized on lysosomes was significantly lower in the 2-BP-treated group compared to the control group (Fig. 4H–J). Collectively, these findings suggest that ZDHHC9 palmitoylates LAMTOR1, thereby enhancing its capacity to recruit mTOR to lysosomes.

### ZDHHC9 palmitoylates LAMTOR at Cys3 and Cys4

**Fig. 5:**
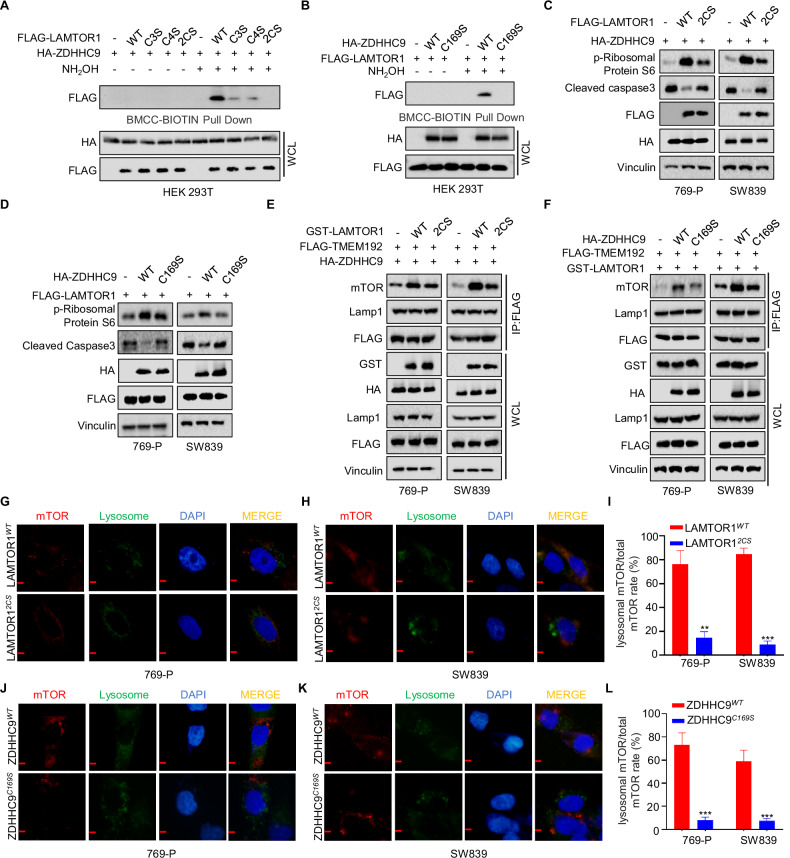
ZDHHC9 palmitoylates LAMTOR at Cys3 and Cys4. **A** HEK-293T cells were transfected with HA-ZDHHC9 and FLAG-LAMTOR1^*WT*^ or mutated FLAG-LAMTOR1. The cell lysates were subjected to conduct to ABE assays. **B** HEK-293T cells were transfected with FLAG-LAMTOR1 and HA-ZDHHC9^*WT*^ or HA-ZDHHC9^*C169S*^. The cell lysates were subjected to conduct to ABE assays. **C** 769-P and SW839 cells transfected with HA-ZDHHC9 and FLAG-LAMTOR1^*WT*^ or FLAG-LAMTOR1^*2CS*^ were subjected to detect the level of p-Ribosomal Protein S6 and Cleaved Caspase3. **D** 769-P and SW839 cells transfected with FLAG-LAMTOR1 and HA-ZDHHC9^*WT*^ or HA-ZDHHC9^*C169S*^ were subjected to detect the level of p-Ribosomal Protein S6 and Cleaved Caspase3. **E** 769-P and SW839 cells transfected with the FLAG-TMEM192, HA-ZDHHC9, GST-LAMTOR1^*WT*^ or GST-LAMTOR1^*2CS*^ were subjected to immunoprecipitation with anti-FLAG. **F** 769-P and SW839 cells transfected with the FLAG-TMEM192, GST-LAMTOR1, HA-ZDHHC9^*WT*^ or HA-ZDHHC9^*C169S*^ were subjected to immunoprecipitation with anti-FLAG. **G**–**I** Confocal immunofluorescence microscopic analysis of mTOR and lysosome in 769-P and SW839 cells transfected with FLAG-LAMTOR1^*WT*^ or FLAG-LAMTOR1 ^*2CS*^. (Scale bar, 50 µm). ***p < 0.001. **J**–**L** Confocal immunofluorescence microscopic analysis of mTOR and lysosome in 769-P and SW839 cells transfected with HA-ZDHHC9^*WT*^ or HA-ZDHHC9^*C169S*^. (Scale bar, 50 µm). ***p < 0.001. All data are presented as mean ± SD of three independent experiments.

All reported palmitoylation events occur on the cysteine (Cys) residues of the substrate. To determine which specific cysteine residue is involved in ZDHHC9-mediated LAMTOR1 palmitoylation, we mutated Cys residues of LAMTOR1 to serine (Ser). As demonstrated in the ABE experiment shown in Fig. 5A, mutating either Cys3 or Cys4 alone did not completely abolish ZDHHC9-mediated LAMTOR1 palmitoylation. Only the simultaneous mutation of both Cys3 and Cys4 eliminated LAMTOR1 palmitoylation. Consistent with these findings, previous studies have indicated that Cys169 is the functional site responsible for the enzymatic catalytic activity of ZDHHC9 [24]. As illustrated in Fig. 5B, mutating the 169 residues of ZDHHC9 from cysteine (Cys) to serine (Ser) abolished its ability to palmitoylate LAMTOR1. Subsequent western blotting analyses revealed that either mutating the palmitoylation sites of LAMTOR1 or inactivating the catalytic site of ZDHHC9 suppressed the overexpression-induced upregulation of p-S6K and downregulation of Cleaved Caspase-3 (Fig. 5C, D). Furthermore, lysosome immunoprecipitation (IP) experiments demonstrated that these mutations impaired the recruitment of LAMTOR1 to mTOR (Fig. 5E, F). Consistent with these findings, immunofluorescence results corroborated the data obtained from lysosome IP experiments (Fig. 5G–L). Collectively, these experiments indicate that the Cys169 residue of ZDHHC9 is critical for catalyzing the palmitoylation of LAMTOR1 at its Cys3/4 sites, thereby positively regulating the mTOR signaling pathway.

### ZDHHC9 knock-down combined with rapamycin restrains RCC growth in vitro and in vivo

**Fig. 6:**
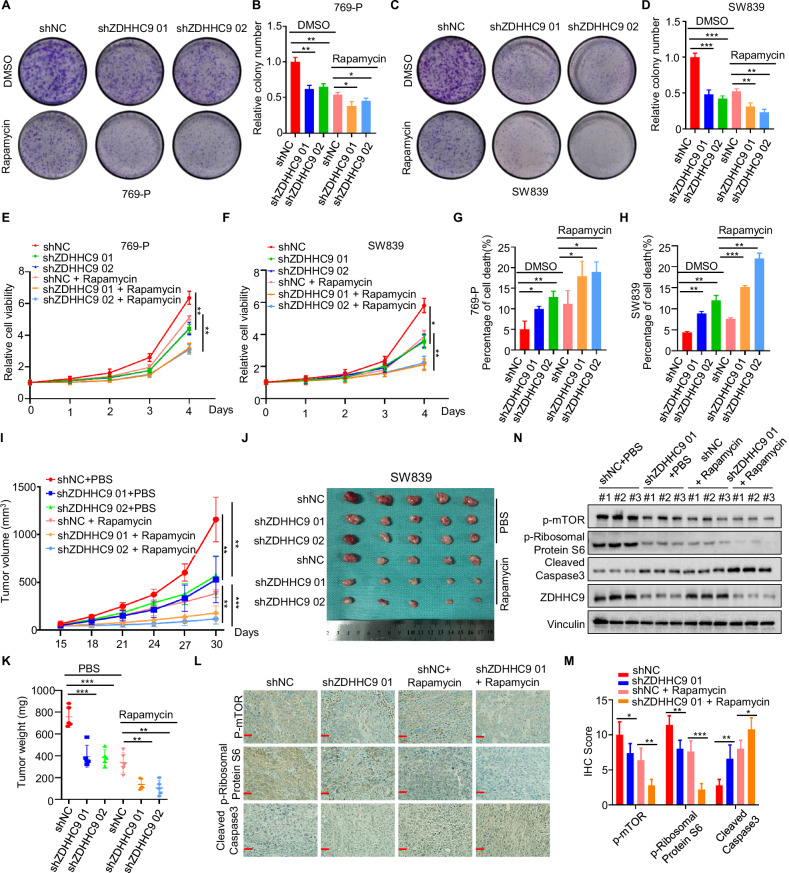
ZDHHC9 knock-down combined with rapamycin restrains RCC growth in vitro and in vivo. **A**–**D** Colony formation assays and quantification results of 769-P and SW839 shNC/shZDHHC9 cells treated with or without Rapamycin (20 μM; 24 h). *p < 0.05, **p < 0.01. **E**, **F** The growth curves of 769-P and SW839 shNC/shZDHHC9 cells treated with or without Rapamycin (20 μM; 24 h). ***p < 0.001. **G**, **H** Statistic analysis of flow cytometry analysis for detecting apoptotic cells in 769-P and SW839 shNC/shZDHHC9 cells treated with or without Rapamycin (20 μM; 24 h). *p < 0.05. **p < 0.01. ***p < 0.001. **I** The growth curves of xenografts in different groups. Tumor volumes were measured every 3 days from day 15 to day 30. **p < 0.01. After 30 days, the nude mice were sacrificed, **J** illustration of tumors excised from male nude mice in each group, and **K** SW839 shNC/shZDHHC9 xenografts were weighed. **p < 0.01. **L**, **M** Immunohistochemical analysis and quantification results of p-mTOR, p-Ribosomal Protein S6 and Cleaved Caspase3 in tumor xenografts. (Scale bar, 200 µm) *p < 0.05. **p < 0.01. ***p < 0.001. **N** Western blotting analysis of p-mTOR, p-Ribosomal Protein S6 and Cleaved Caspase3 expression in tumor xenografts. All data are presented as mean ± SD of three independent experiments.

To investigate the in vitro inhibitory effect of ZDHHC9 knock-down combined with rapamycin on the proliferation of renal cell carcinoma (RCC), we performed a series of experiments. Plate cloning assays (Fig. 6A–D), MTT experiments (Fig. 6E, F), and flow cytometry analyses (Figs. 6G, H and S4A, B) demonstrated that the combination of ZDHHC9 knock-down and rapamycin exerted a more pronounced inhibitory effect on RCC cell proliferation compared to rapamycin treatment alone. Subsequently, we examined the in vivo inhibitory effect using a subcutaneously implanted tumor model in nude mice inoculated with SW839 shNC/shZDHHC9 cells. When the tumor volume reached approximately 50 mm³, the treatment groups were given Rapamycin (2.0 mg/kg; intraperitoneal injection; once every 3 days; for a total of 6 times), while the control groups were given the same dosing strategy with the same volume of solvent for intraperitoneal injection. The results revealed that both ZDHHC9 knock-down and rapamycin treatment suppressed xenograft growth and weight, while their combination significantly enhanced the inhibition of xenograft growth and weight (Fig. 6I–K). Furthermore, immunohistochemistry (Fig. 6L, M) and western blotting (Fig. 6N) of tumor tissues confirmed that the combination of rapamycin and ZDHHC9 knock-down effectively inhibited the mTOR pathway and induced apoptosis. Collectively, these findings indicate that targeting ZDHHC9 in combination with rapamycin yields superior inhibitory effects on RCC, offering a promising strategy for clinical treatment.

## Discussion

**Fig. 7:**
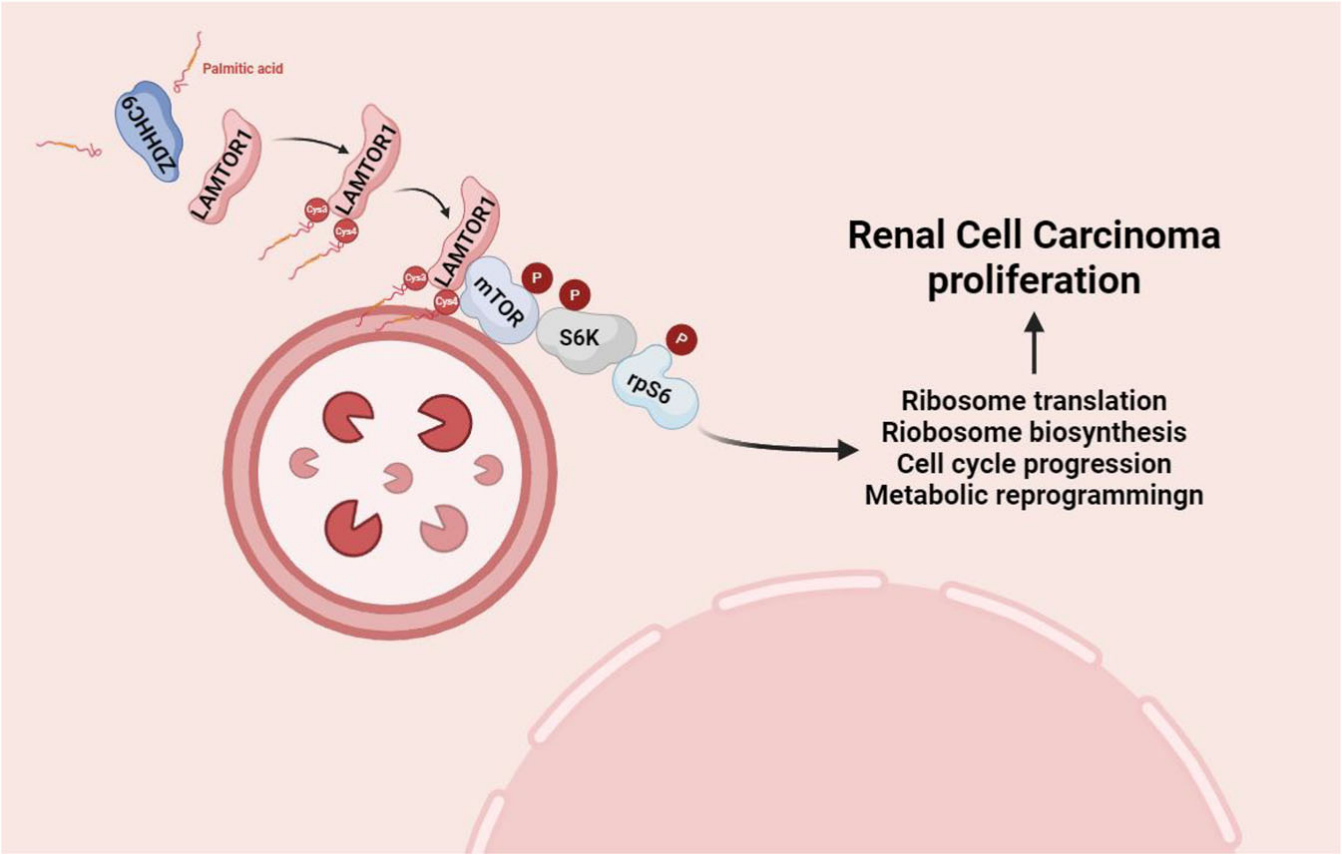
Schematic models. ZDHHC9 exerts its functions via palmitoylating LAMTOR1 to recruit mTOR to lysosome to promote RCC proliferation. Created with bioRender.com.

The mTOR signaling pathway regulates cell proliferation and is over-activation in various types of cancer cells, promoting the malignant progression of carcinoma [34, 35]. Although mTOR inhibitors such as rapamycin have been applied in the treatment of various cancers, as the disease progresses, patients develop varying degrees of drug resistance [36–38]. Therefore, it is exigent to lucubrate the regulatory mechanism of the mTOR signaling pathway to surmount the predicament. This study substantiates that ZDHHC9 interacts with LAMTOR1 and mediates the palmitoylation of LAMTOR1 at its Cys3/4 sites, thereby recruiting mTOR to the lysosomal membrane and activate downstream molecules. Furthermore, immunohistochemical results reveals the high expression levels of both ZDHHC9 and p-Ribosomal Protein S6 in clinical tumor tissue specimens. Meanwhile, we confirm that the combination of mTOR inhibitors rapamycin and ZDHHC9 knock-down markedly constrains tumor progression in mice, suggesting that combine targeting ZDHHC9 and mTOR inhibitors may represent a prominent strategy for RCC treatment (Fig. 7).

ZDHHC9, as one of the members of the ZDHHCx enzyme family that mediate palmitoylation, was overexpression in plentiful malignant tumors and was related to poor prognosis [15, 16, 39, 40]. Our immunohistochemistry and western blotting experiments confirmed that ZDHHC9 was highly expressed in RCC tissues. Moreover, the plate cloning, EdU staining and flow cytometry assays substantiated that ZDHHC9 promoted the proliferation of RCC. The leukemia mice with *ZDHHC9*^*KO*^ presents a preferable survival than the mice leukemia with *ZDHHC9*^*WT*^ [41]. Compared with the subcutaneous gliomas of *ZDHHC9*^*WT*^, those of *ZDHHC9*^*C169S*^ or *ZDHHC9*^*KO*^ emerged the similar milder malignancy in mice [24]. The palmitoylation of PD-L1 mediated by ZDHHC9 enabled breast cancer and lung adenocarcinoma to evade immune surveillance [42, 43]. Moreover, targeting ZDHHC9 could enhance the immunotherapy efficacy of colorectal carcinoma, pancreatic carcinoma and triple-negative breast cancer (TNBC) [16, 39, 44]. It was substantiated that ZDHHC9 was involved in the Gemcitabine and Cisplatin resistance to bladder cancer and containing ZDHHC9 increased the sensitivity of bladder cancer to Gemcitabine [15]. Not only that, impairing the palmitoylation of LDHA catalyzed by ZDHHC9 improved the sensitivity of pancreatic cancer to Doxorubicin [39]. We verified that ZDHHC9 interacted with LAMTOR1 via its DHHC domain through immunoprecipitation experiments. Then we found ZDHHC9 knock-down whittled the activation of mTOR signaling pathway regulated by LAMTOR1. In addition, we confirmed that ZDHHC9 meditated palmitoylation of LMATOR1 at its Cys3/4 sites by the ABE assay.

Plentiful studies confirmed that LAMTOR1/2/3 complex is necessary for activating mTROC1 and downstream molecules [45, 46]. LAMTOR1 emerged higher level in urinary exosomes from prostate cancer patients compared to healthy male and was also highly expressed in hepatoma carcinoma tissues and induced its malignant metabolic changes [47, 48]. Targeting LAMTOR1 delayed the process of inflammatory tissues transforming into cancerous tissues [49]. In addition, inhibition of LAMTOR1 enhanced the killing ability of rapamycin on Hela cells [50]. Meanwhile, consistent with ZDHHC9, LAMTOR1 also promoted the escape of cancer cells from immune surveillance [51]. Suppressing the expression of LAMTOR1 led to the inability of mTOR to be localized on the lysosomal membrane and crippled the cells proliferation [46, 52]. Enhancing the function of LAMTOR1 contributed the recruitment of mTOR to lysosomes, thereby resulting in intensive resistance to Docetaxel and aggravated hepatic metastases of TNBC [53]. What’s more, the enhanced LAMTOR1 meditated by chemotherapy led to an excessive degradation of cGAS, resulting in drugs-resistance. While, silencing LAMTOR1 elevated the immunotherapy and overcame chemotherapy resistance [54]. Currently, a literature reported that the de-palmitoylase PPT1 regulated the accumulation of palmitoylated proteins to influence the mTOR and lysosomal catabolic processes [55]. However, the substrate proteins affected by PPT1 have not been revealed. In the future, we will explore whether PPT1 can regulate the progression of RCC by reducing the LAMTOR1 palmitoylation. In present study, we first demonstrated that ZDHHC9 regulates mTOR by catalyzing LAMTOR1 palmitoylation at its Cys3/4 sites. Suppressing ZDHHC9 in combination with the mTOR inhibitor rapamycin exerted a more potent inhibitory effect on cell proliferation and tumor growth in mice than either ZDHHC9 suppression alone or rapamycin monotherapy.

In conclusion, this research revealed the significant role of ZDHHC9 in RCC. The underlying mechanism that ZDHHC9 activities mTOR pathway via palmitoylating LAMTOR1 therefore promoting its ability to recruit mTOR sustains our findings. Taken together, our study indicates that the combination of restraining ZDHHC9 and mTOR inhibitor rapamycin will become a novel strategy in RCC.

## Supplementary information

Supplementary manuscript

Merge Western blot

## Data Availability

The data that support the findings of this study are available from the corresponding author upon reasonable request.
