# Supplementary material for: ZDHHC9 palmitoylates LAMTOR1 to promote renal cell carcinoma malignant progression
Source: Cell Death Dis. 2026 Mar 19;17(1):323. doi: 10.1038/s41419-026-08558-8 (PMC13039863; doi:10.1038/s41419-026-08558-8)
Supplement: Supplementary file 1 — Supplementary manuscript [file 41419_2026_8558_MOESM1_ESM.doc]

Supplementary Figures for

**ZDHHC9 palmitoylates LAMTOR1 to promote renal cell carcinoma malignant progression**

Bo Liu^1,2,#^, Tao Hou^3,#^, Xizhi Liu^1^, Lu Liu^1,4^, Zhiqiang Ma^5,*^, Yujiao Zhang^1,*^

^1^ Department of Respiratory and Critical Care Medicine, The Second Affiliated Hospital of Xi'an Jiaotong University, Xi'an, 710004, Shaanxi, China.

^2^ Department of Urology, The First Affiliated Hospital of Xi'an Jiaotong University, Xi'an, 710061, Shaanxi, China.

^3^ Department of Pathology, Beth Israel Deaconess Medical Center, Harvard Medical School, Boston, 02215, MA, USA.

^4^ Department of Respiratory and Critical Care Medicine, Xi'an Aerospace General Hospital, Xi'an, 710100, Shaanxi, China.

^5^ Department of Medical Oncology, Senior Department of Oncology, Chinese PLA General Hospital, The Fifth Medical Center, 100853, Beijing, China.

^#^ These authors contributed equally to this work and should be considered co-first authors.

*Correspondence:

Dr. Zhiqiang Ma, Department of Medical Oncology, Senior Department of Oncology, Chinese PLA General Hospital, The Fifth Medical Center, Beijing 100853, P.R. China. E-mail: mazhiqiang@301hospital.com.cn

Dr. Yujiao Zhang, Department of Respiratory and Critical Care Medicine, The Second Affiliated Hospital of Xi'an Jiaotong University, 157 West Fifth Road, Xi'an, 710004, Shaanxi, China. E-mail: yujiaozhang_123@163.com

**This file includes:**

Supplementary Figures 1-3

Supplementary Table 1


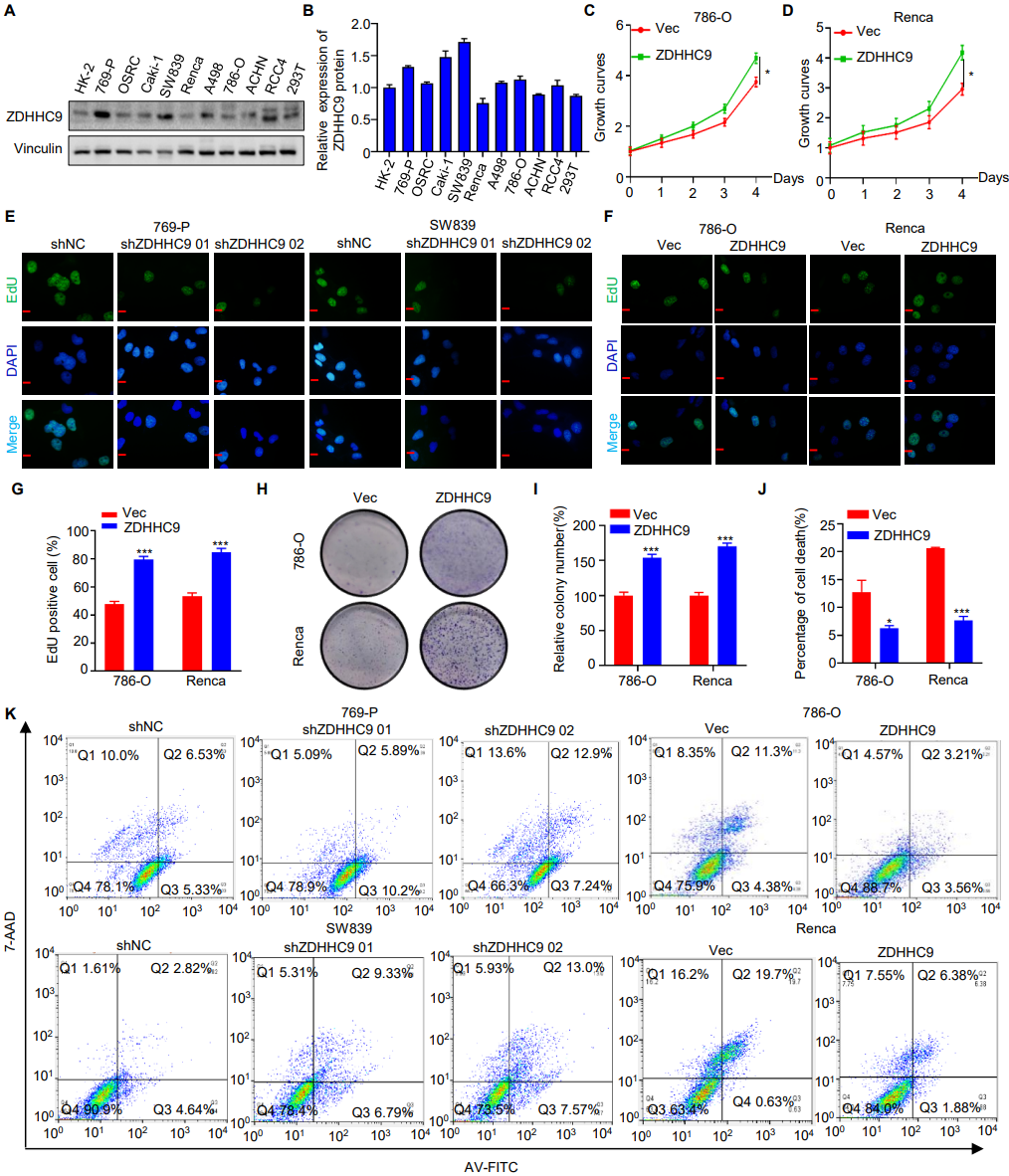


**Figure S1 ZDHHC9 is overexpressed and acts as an oncogene in RCC.** **A-B** Western blotting analysis and quantification results of ZDHHC9 expression in the normal human renal cell line and RCC cell lines. **C-D** MTT assay was performed to detect the cell viability in 786-O and Renca with ZDHHC9 overexpression. **P*<0.05. **E** EdU assay was performed to detect proliferation in 769-P and SW839 cells with ZDHHC9 knock-down and **F** 786-O and Renca cells with ZDHHC9 overexpression. **G** Statistic analysis of (**F**). ****P*<0.001. **H-I** Colony formation assays and quantification results of 786-O and Renca cells treated with ZDHHC9 overexpression. ****P*<0.001. **J** Statistical analysis of flow cytometry analysis for detecting apoptotic cells in 786-O and Renca cells with ZDHHC9 overexpression. **P*<0.05. ****P*<0.001. **K** Flow cytometry analysis for detecting apoptotic cells in 769-P and SW839 cells with ZDHHC9 knock-down and 786-O and Renca cells with ZDHHC9 overexpression. All data are presented as mean ± SD of three independent experiments.


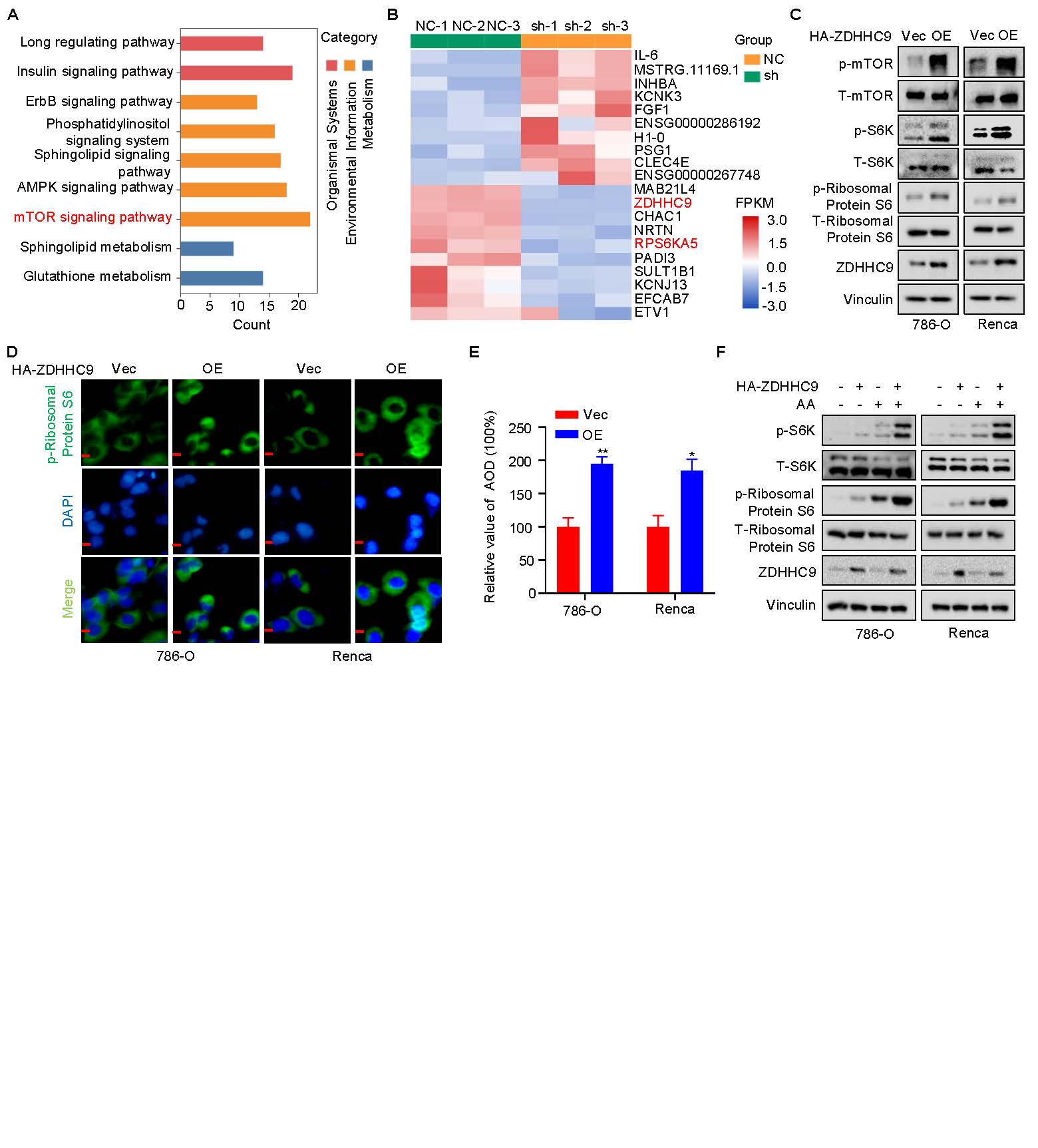
**Figure S2 ZDHHC9 activates the mTOR signaling pathway in RCC. A** RNA sequencing KEGG enrichment results suggested that ZDHHC9 knock-down affected mTOR signaling pathway. **B** Top up and down gene changes in RNA sequencing. **C** Western blotting assay of p-mTOR, T-mTOR, p-S6, T-S6, p-Ribosomal Protein S6, T-Ribosomal Protein S6 and ZDHHC9 in 786-O and Renca cells with ZDHHC9 overexpression. **D** Immunofluorescence assays that performed to detect expression of p-Ribosomal Protein S6 in 786-O and Renca cells with ZDHHC9 overexpression. (Scale bar, 200 µm). **E** Statistic analysis of (**D**). **P*<0.05. ***P*<0.01. **F** 786-O and Renca Vec/OE ZDHHC9 cells were subjected to amino acid starvation for 6 hours and re-supplementation for 1 h. p-S6, T-S6, p-Ribosomal Protein S6, T-Ribosomal Protein S6 and ZDHHC9 were detected.


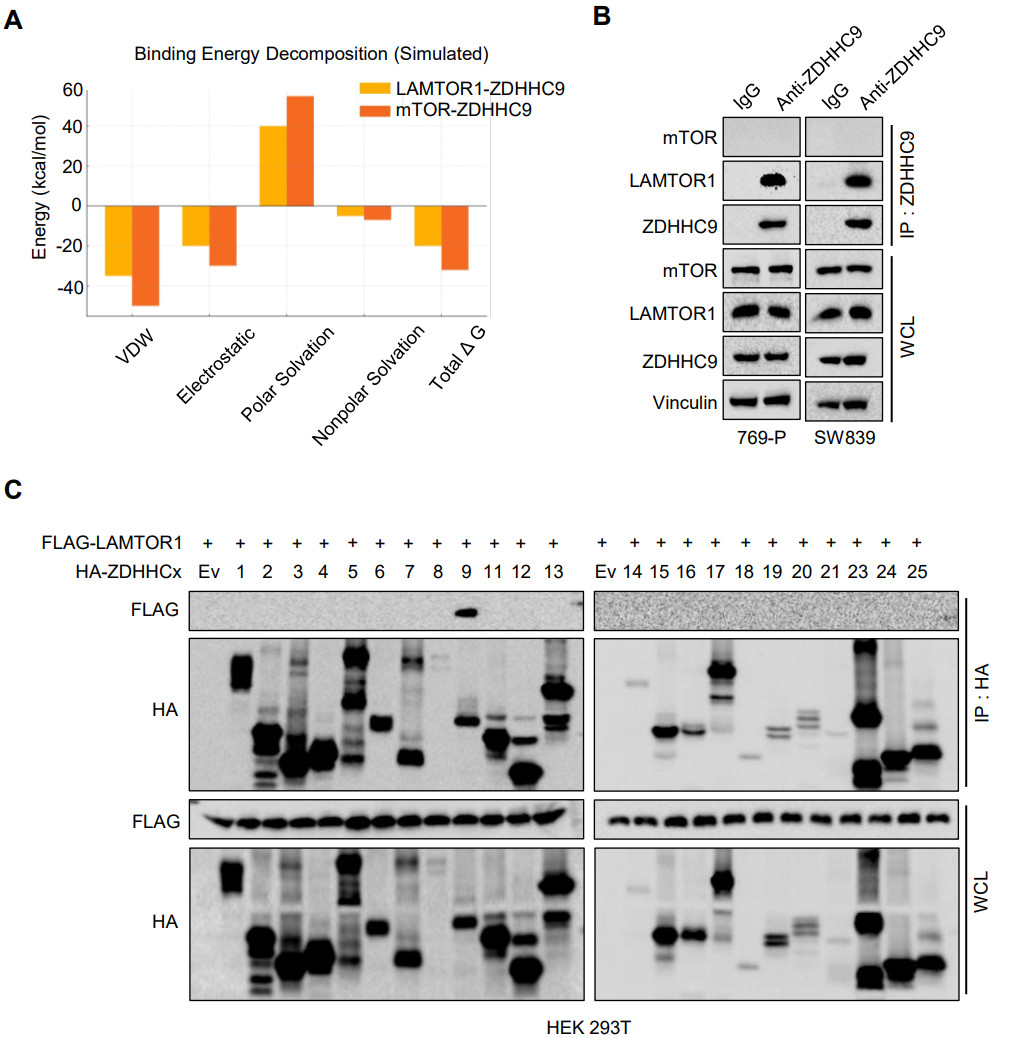


**Figure S3 ZDHHC9 activates the mTOR signaling pathway in RCC. A** The binding energy of the molecular interaction between LAMTOR1-ZDHHC9 and mTOR-ZDHHC9. **B** Western blotting assay endogenous interaction of ZDHHC9, LAMTOR1 and mTOR in 769-P and SW839 cells. **C** Immunoprecipitation experiment on LAMTOR1 with all the enzymes in the ZDHHCx family.


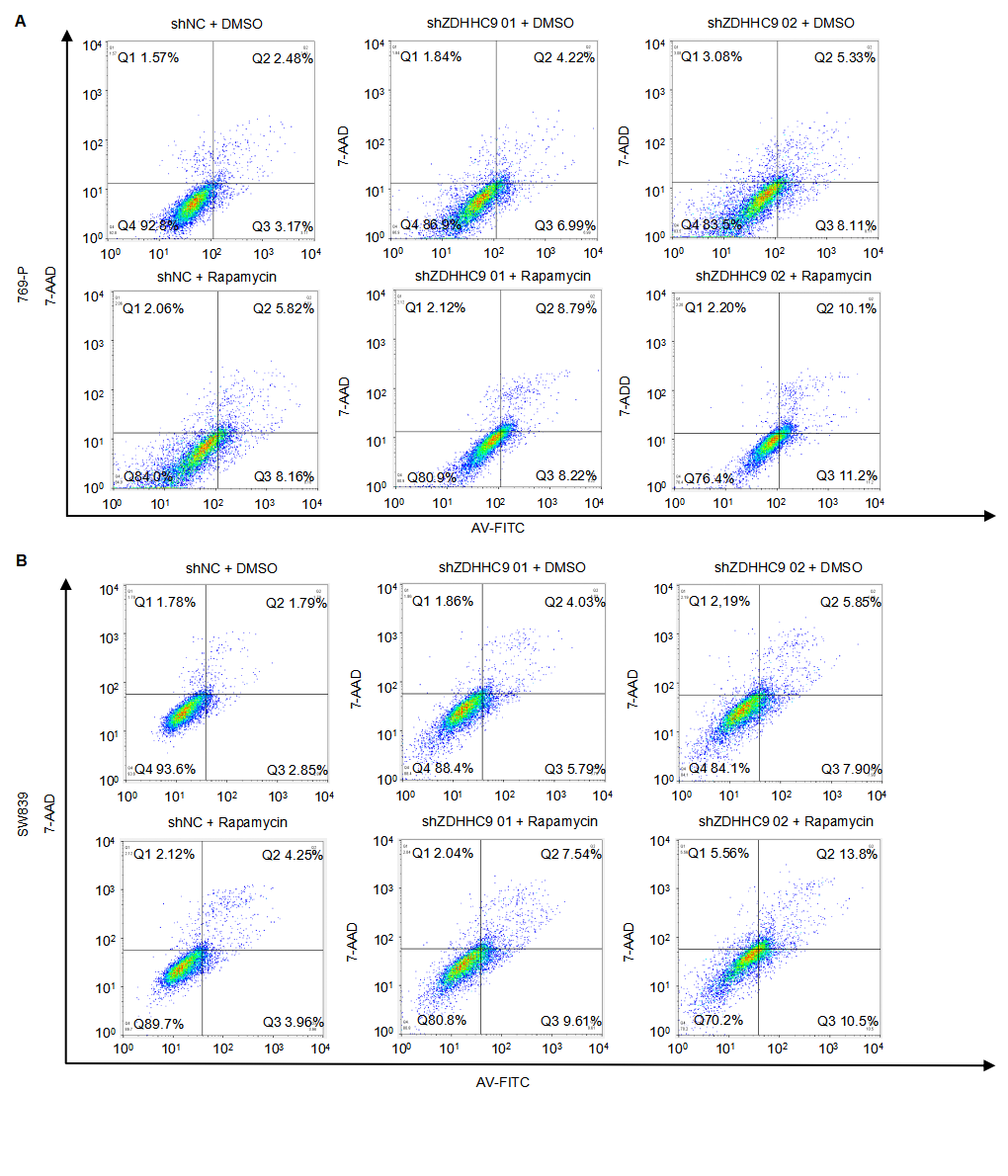


**Figure S4 ZDHHC9 knock-down combined with rapamycin restrains RCC growth in vitro and in vivo. A-B** Flow cytometry analysis for detecting apoptotic cells in 769-P and SW839 shNC/shZDHHC9 cells treated with or without Rapamycin (20 μM).

**Table S1. The sh**RNA sequence.

| ZDHHC9 shRNA sequence-1 | CTGTTACACATGCAAGATCTT |
| --- | --- |
| ZDHHC9 shRNA sequence-2 | GAAGTCCTCATTTGCTTCTTT |
| LAMTOR1 shRNA sequence-1 | CCCATCCCGTTCTCTGATTTG |
| LAMTOR1 shRNA sequence-2 | AGACAGCCAGCAACATCATTG |
