# Supplementary material for: ZDHHC9 palmitoylates LAMTOR1 to promote renal cell carcinoma malignant progression
Source: Cell Death Dis. 2026 Mar 19;17(1):323. doi: 10.1038/s41419-026-08558-8 (PMC13039863; doi:10.1038/s41419-026-08558-8)

Fig. 1D

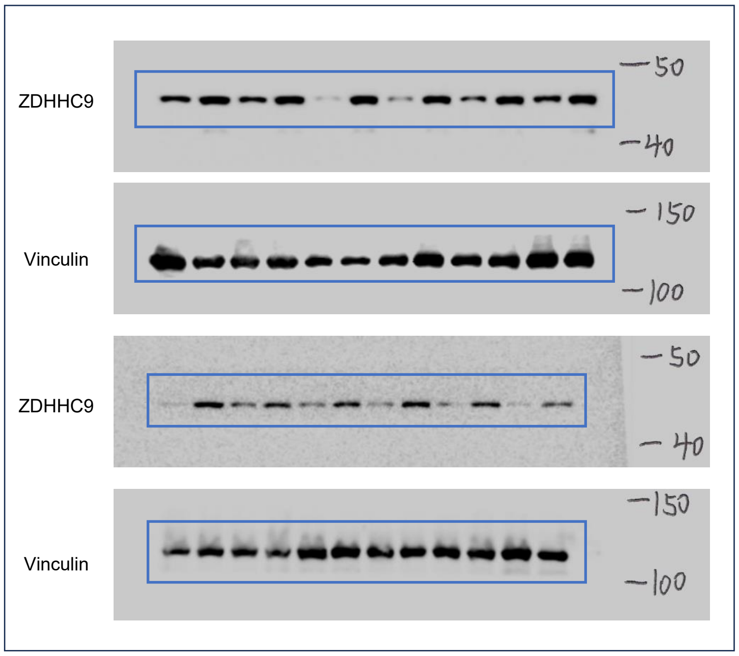

Fig. 1L

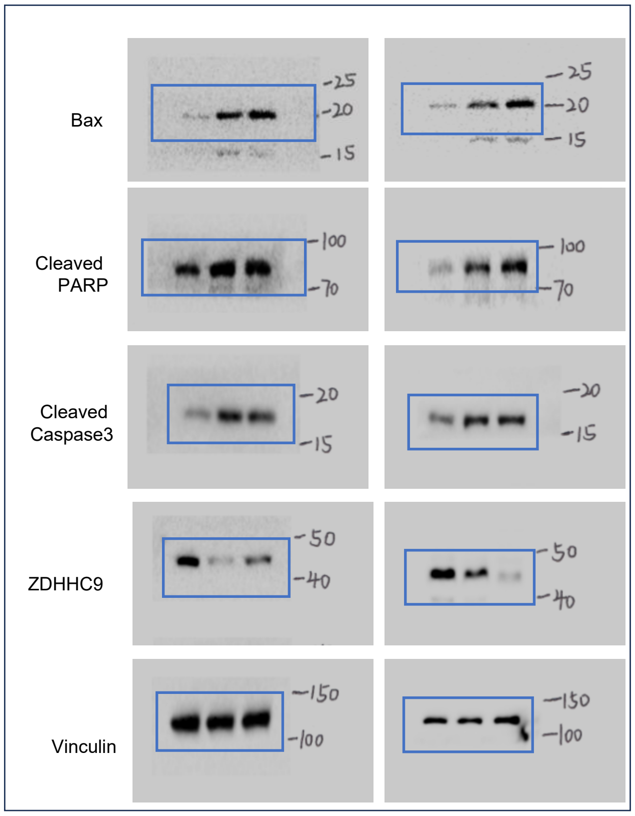

Fig. 1M

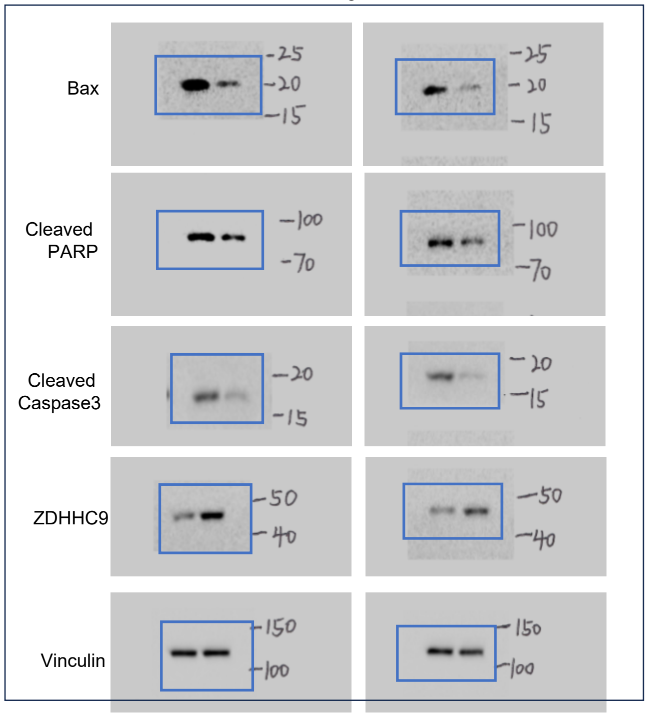

Fig. 1Q

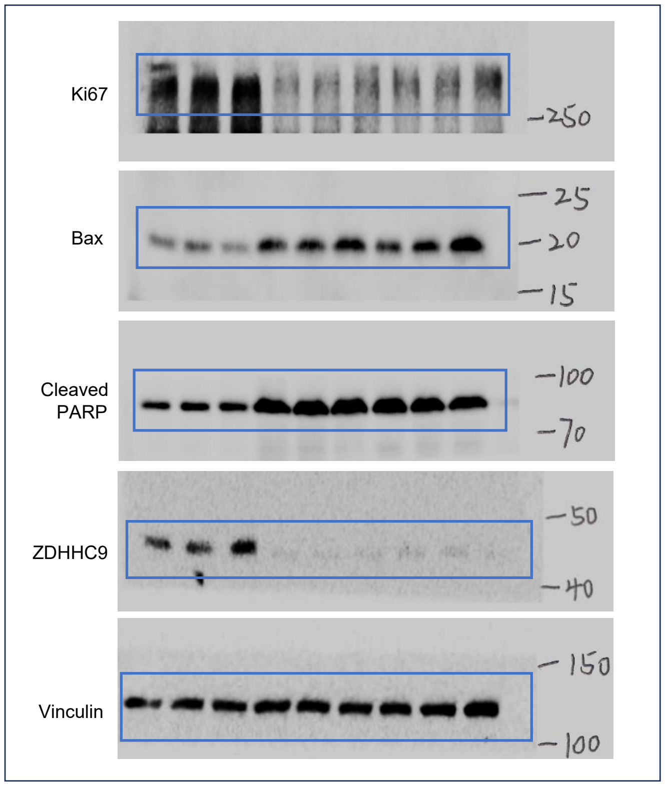

Fig. S1A

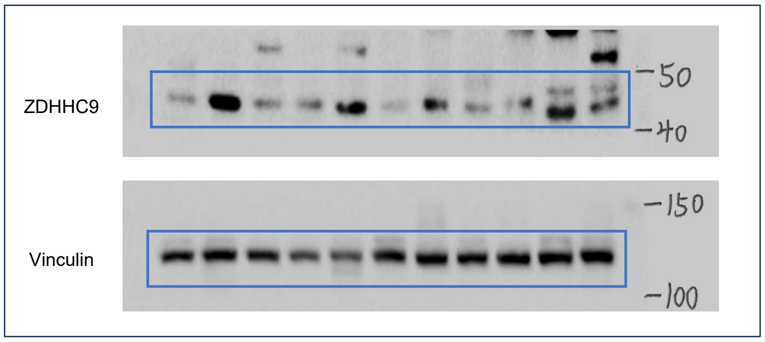

Fig. S2C

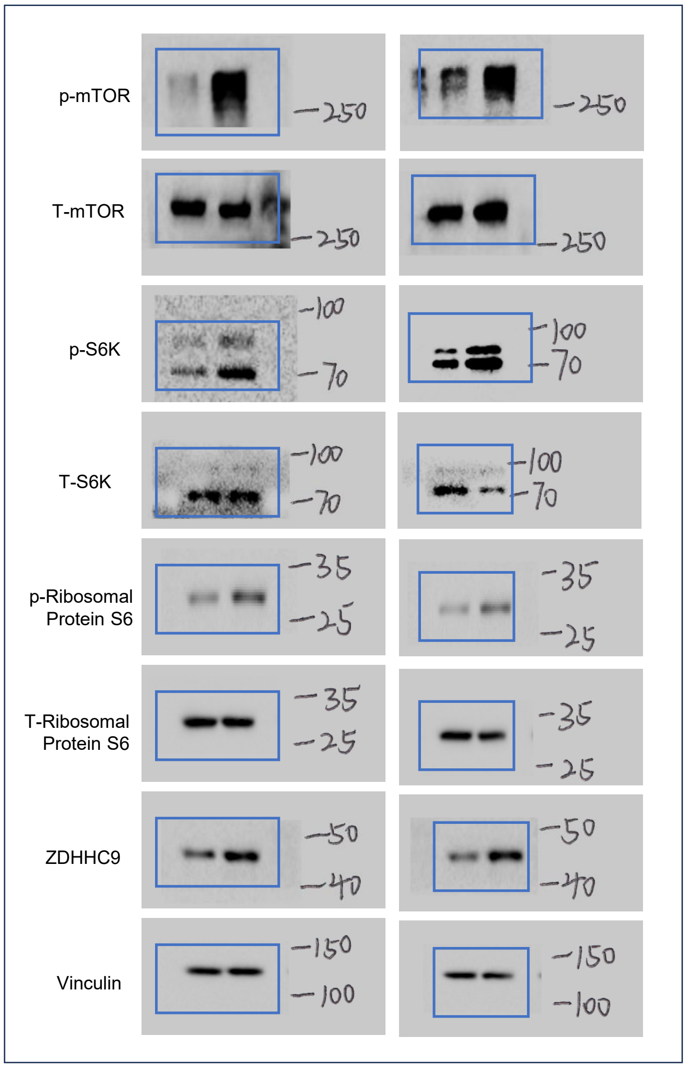

Fig. 2B

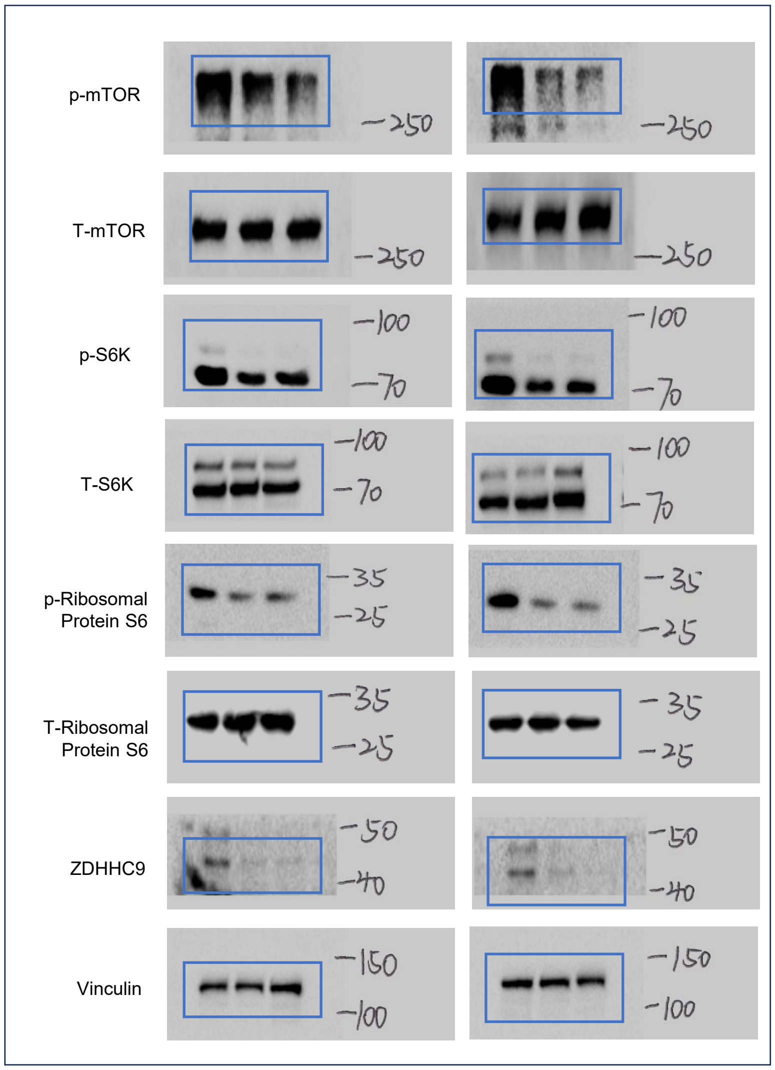

Fig. 2E

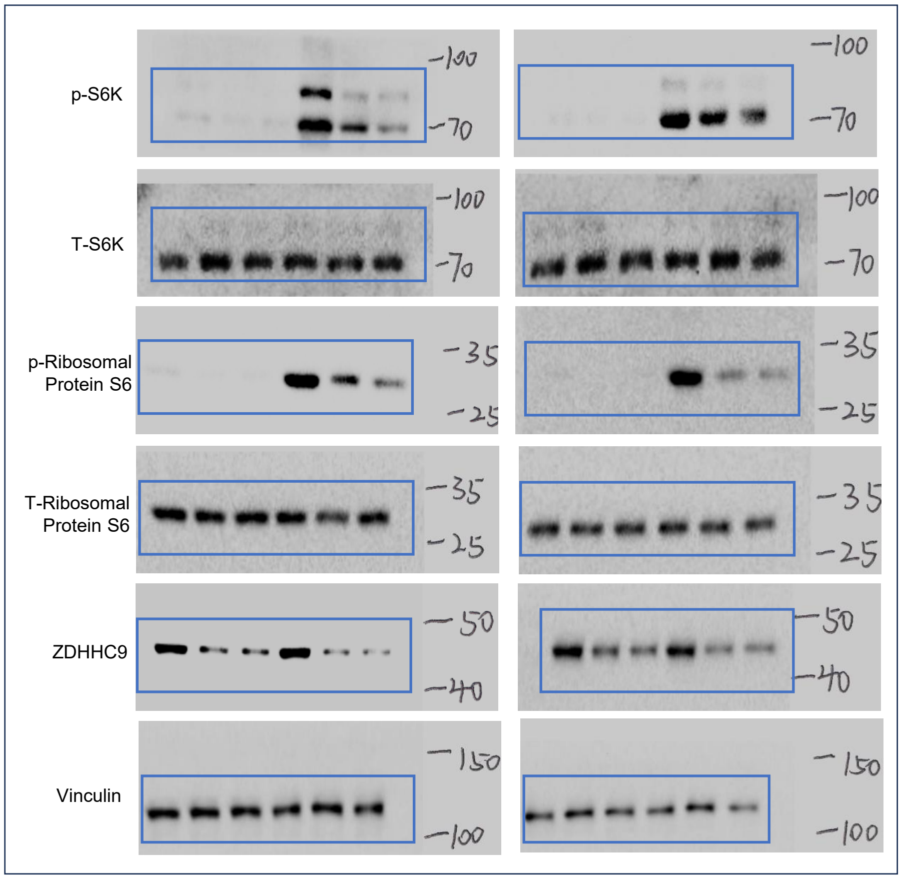

Fig. S2F

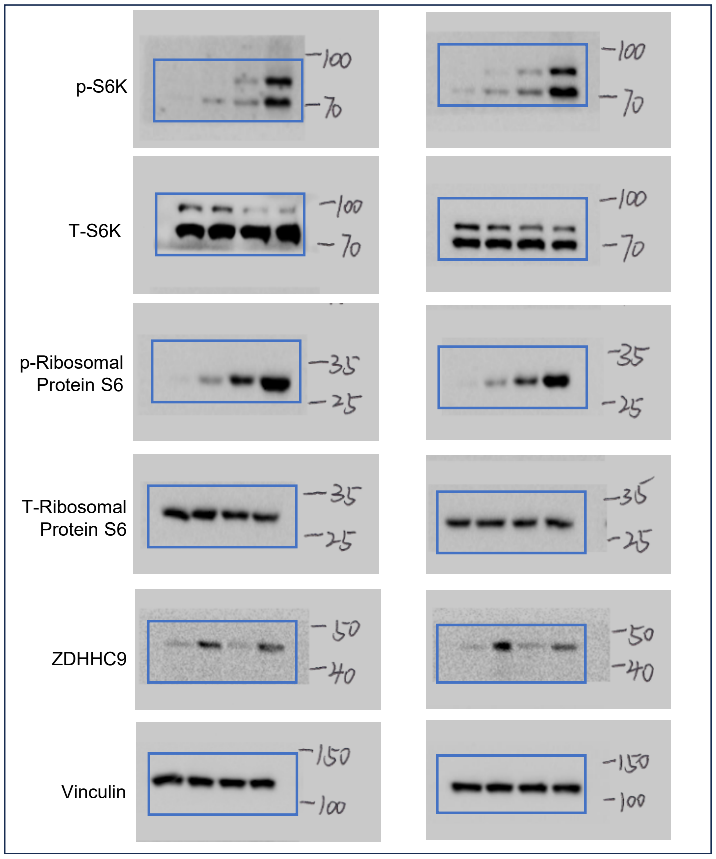

Fig. 2H

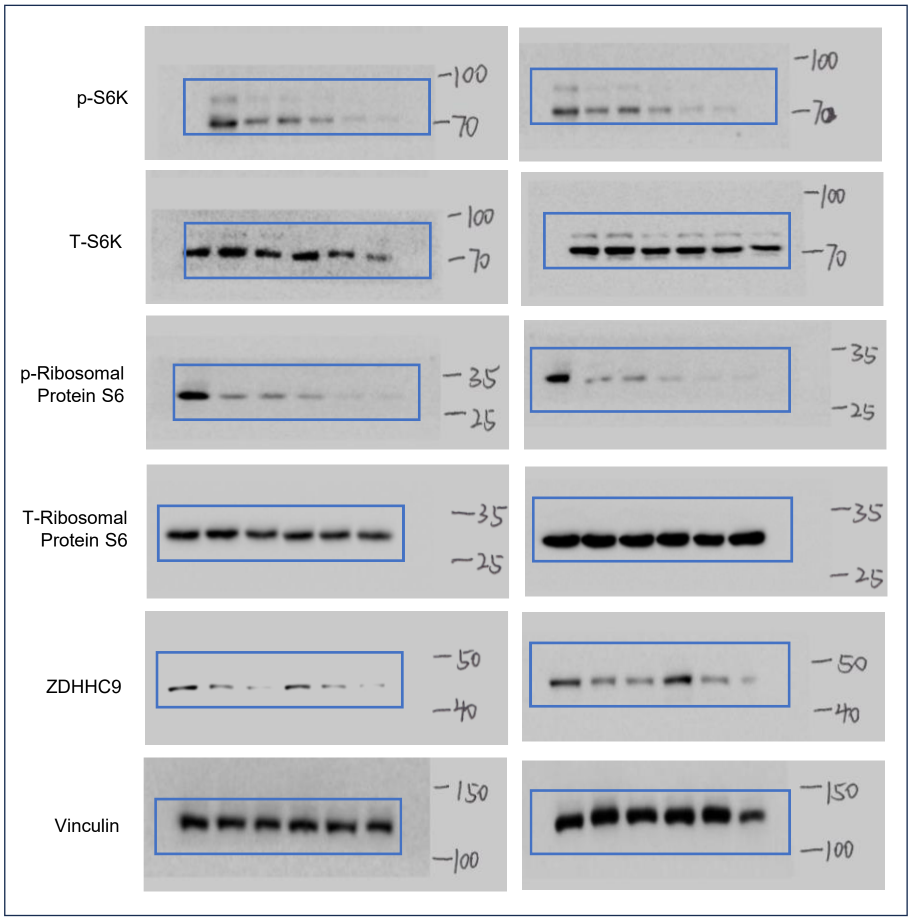

Fig. 2F

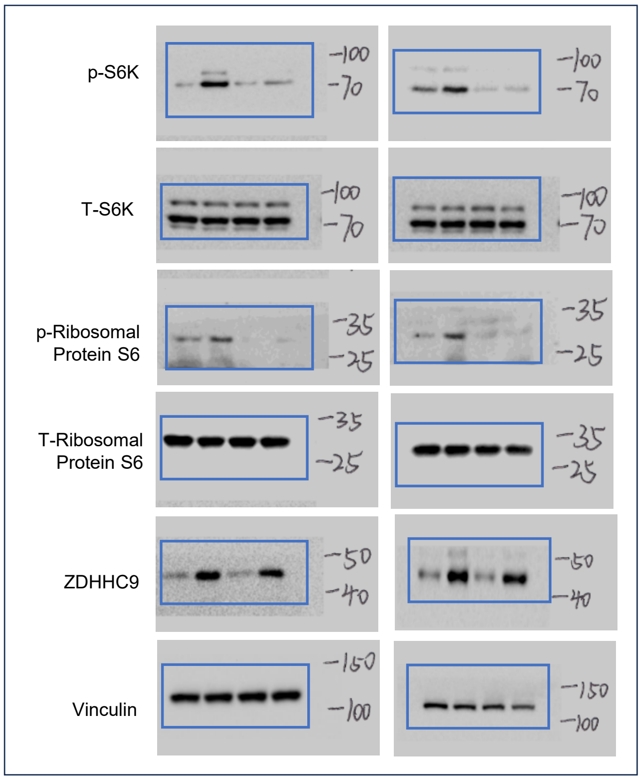

Fig. 2I

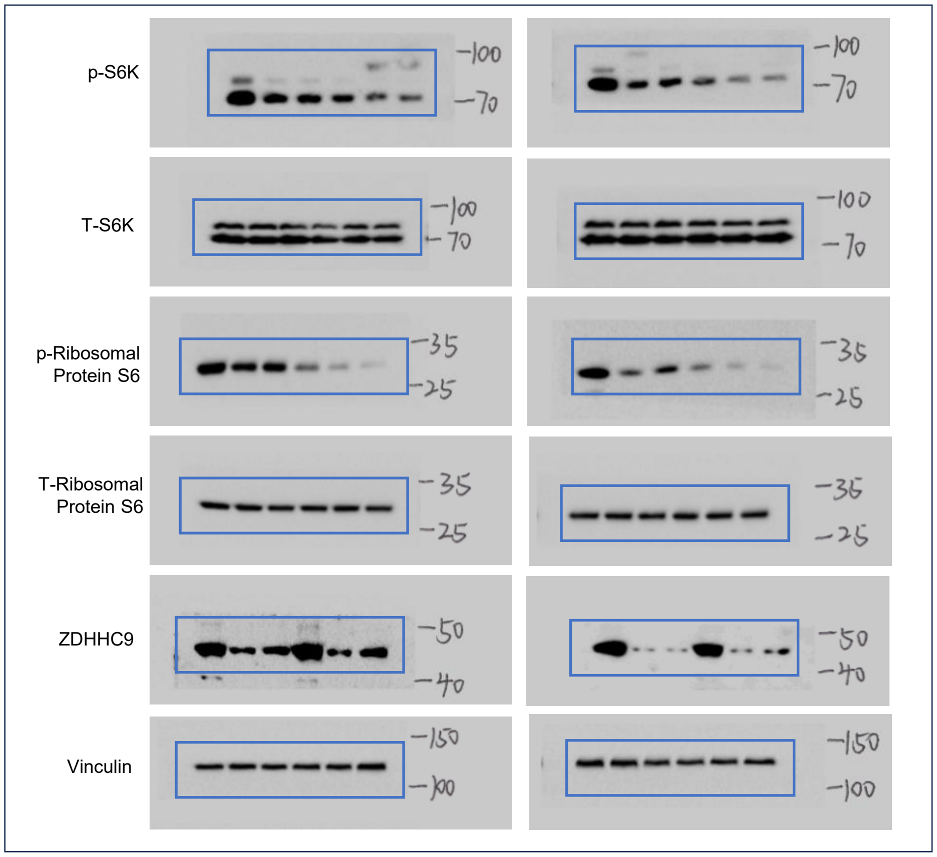

Fig. 2G

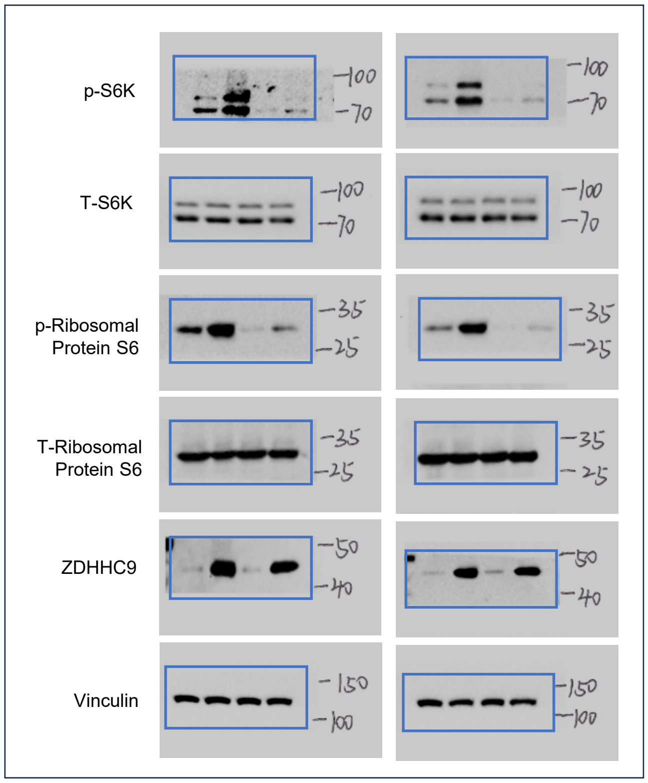

Fig. S3B

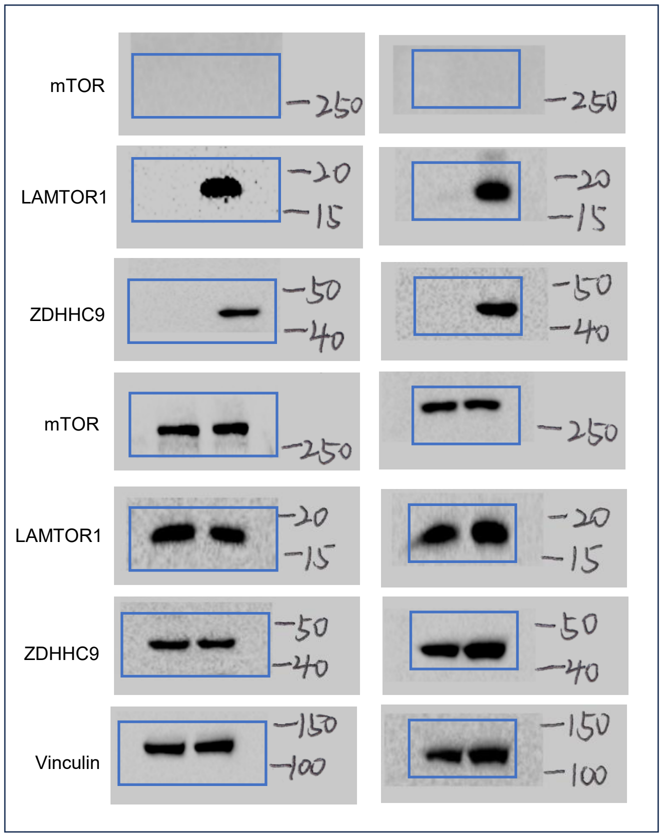

Fig. 3C

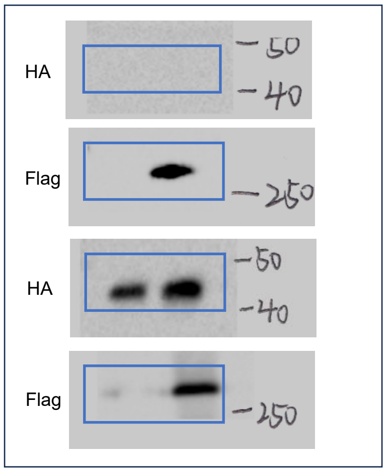

Fig. 3D

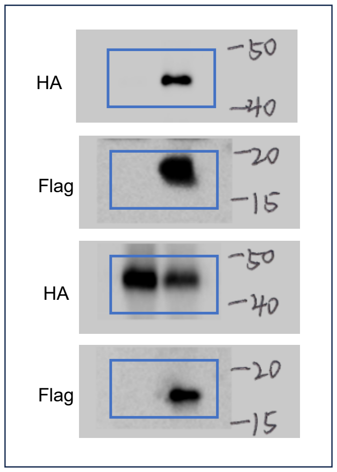

Fig. 3G

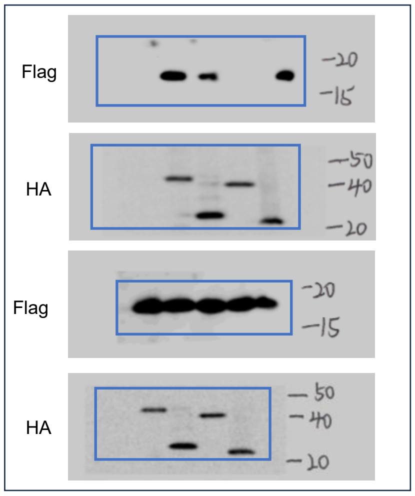

Fig. S3C

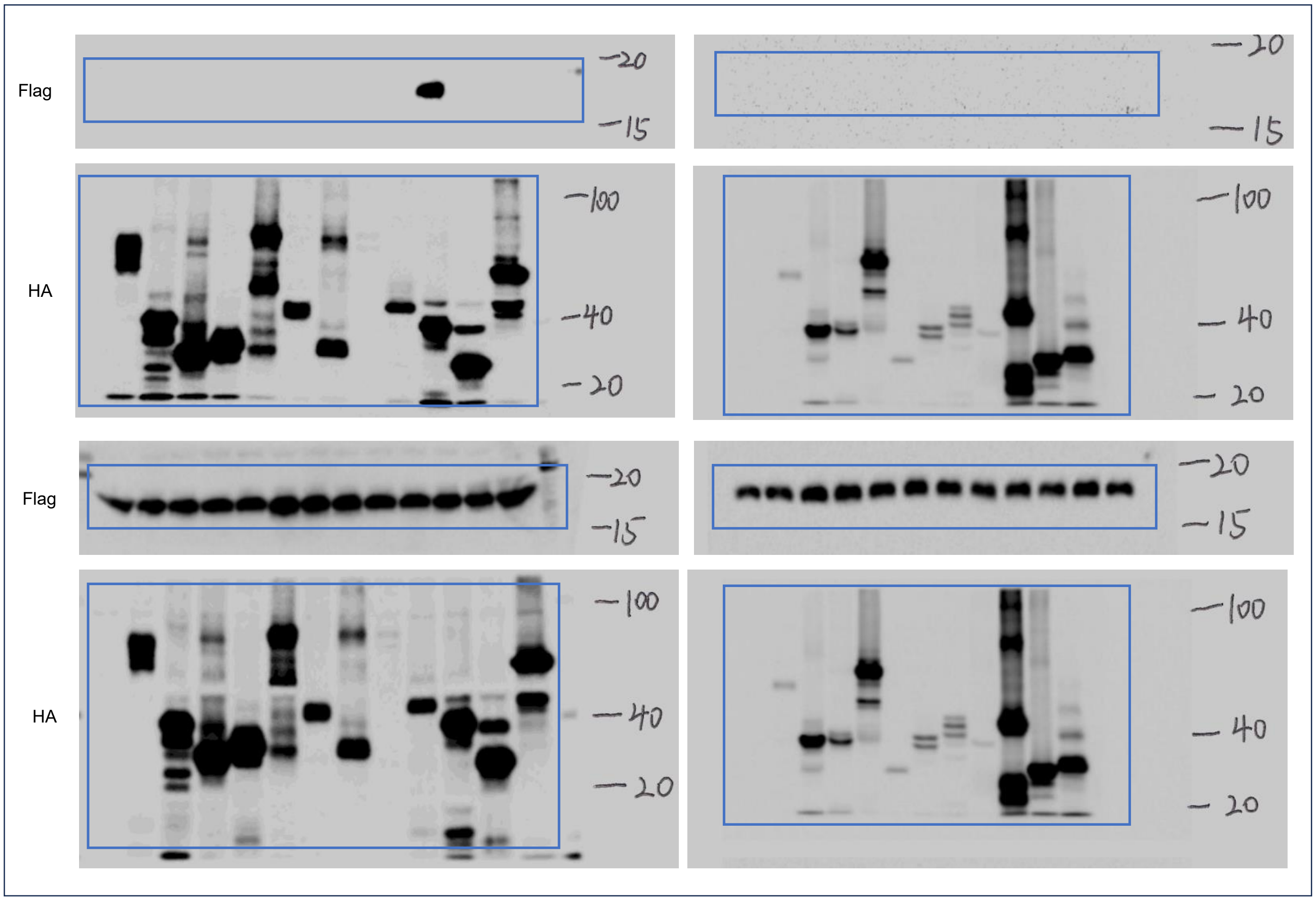

Fig. 3H

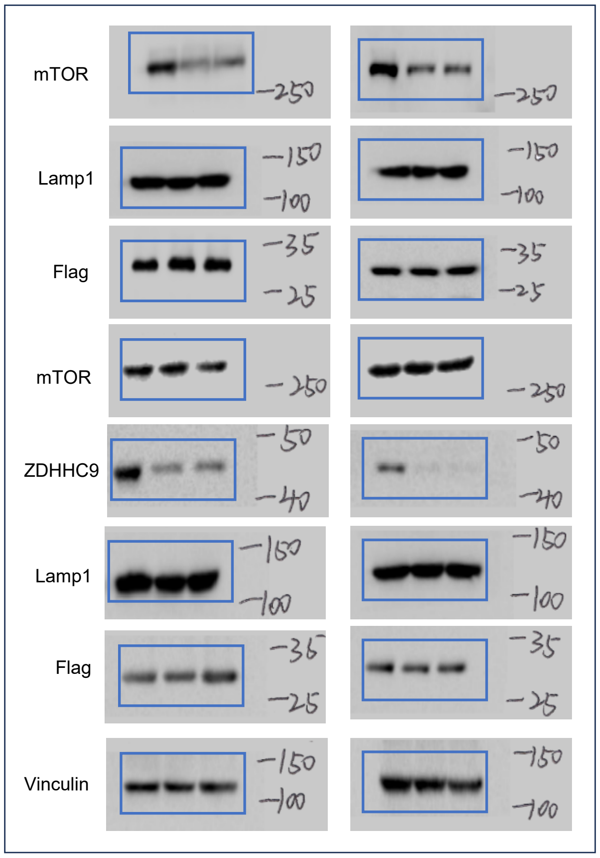

Fig. 3I

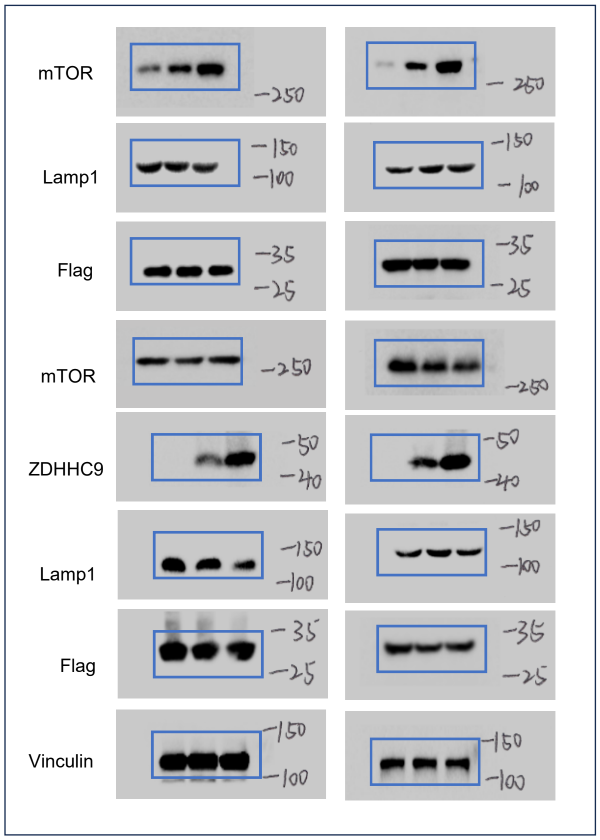

Fig. 3L

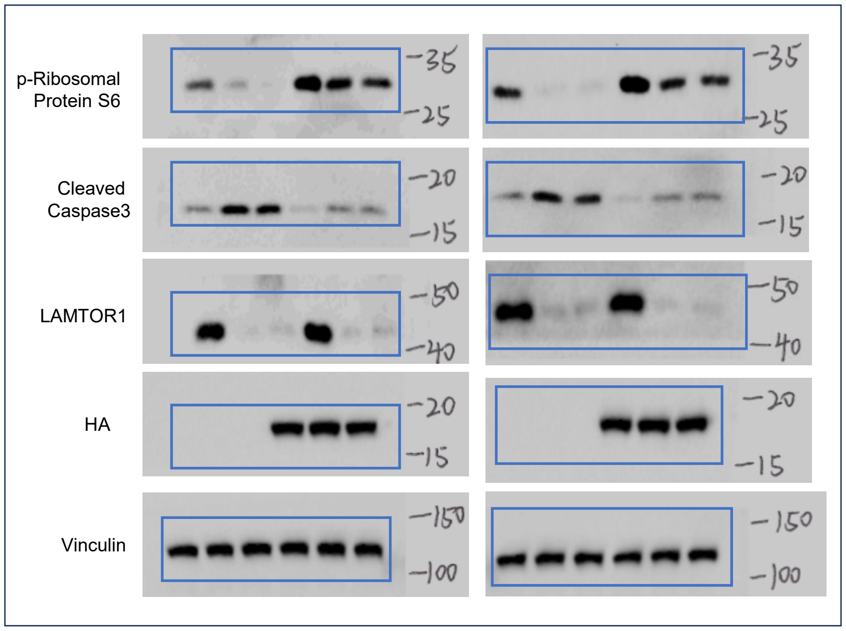

Fig. 3M

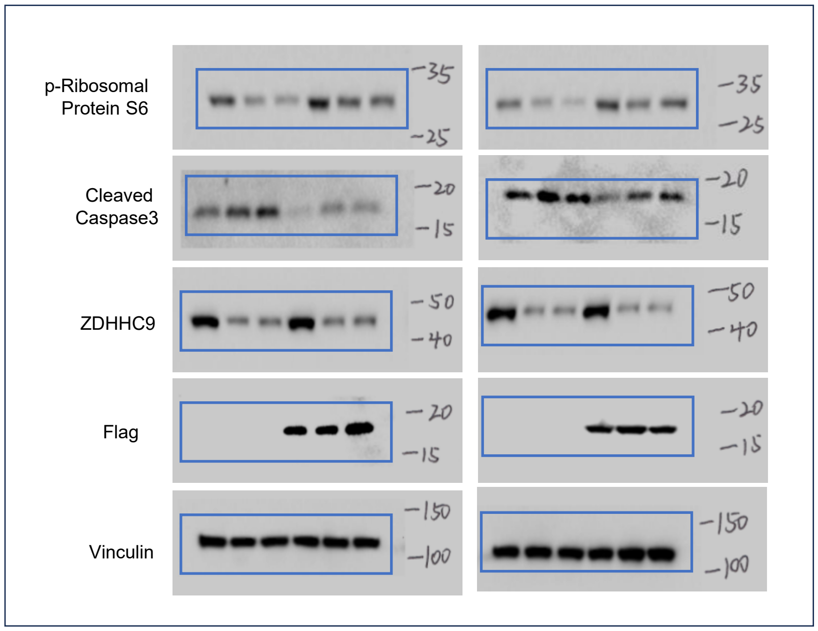

Fig. 4A

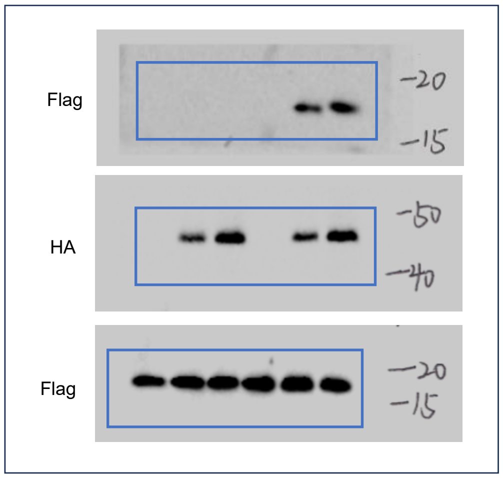

Fig. 4B

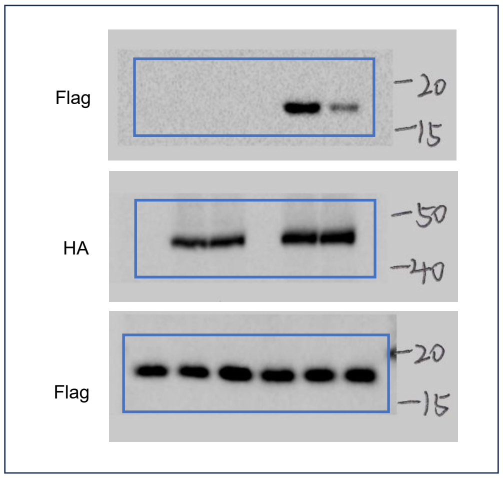

Fig. 4E

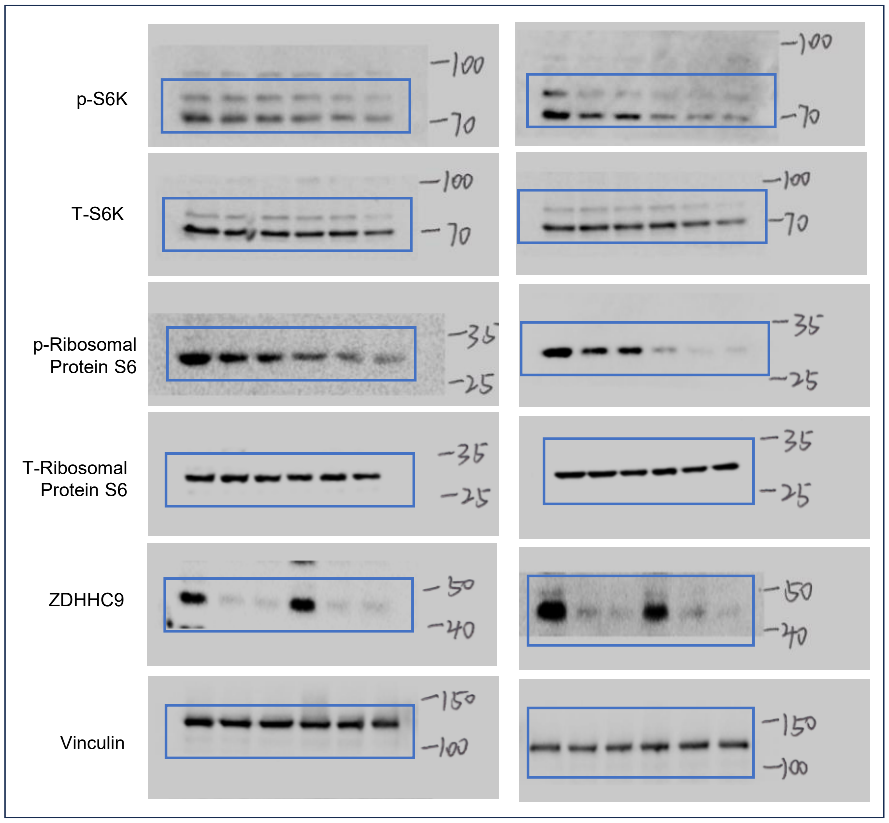

Fig. 4F

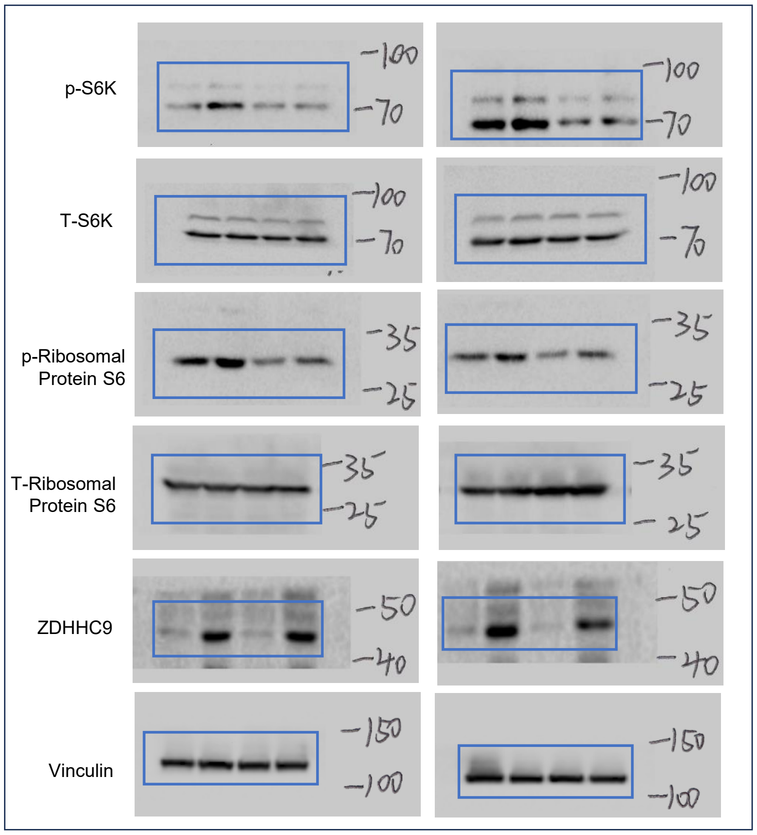

Fig. 4G

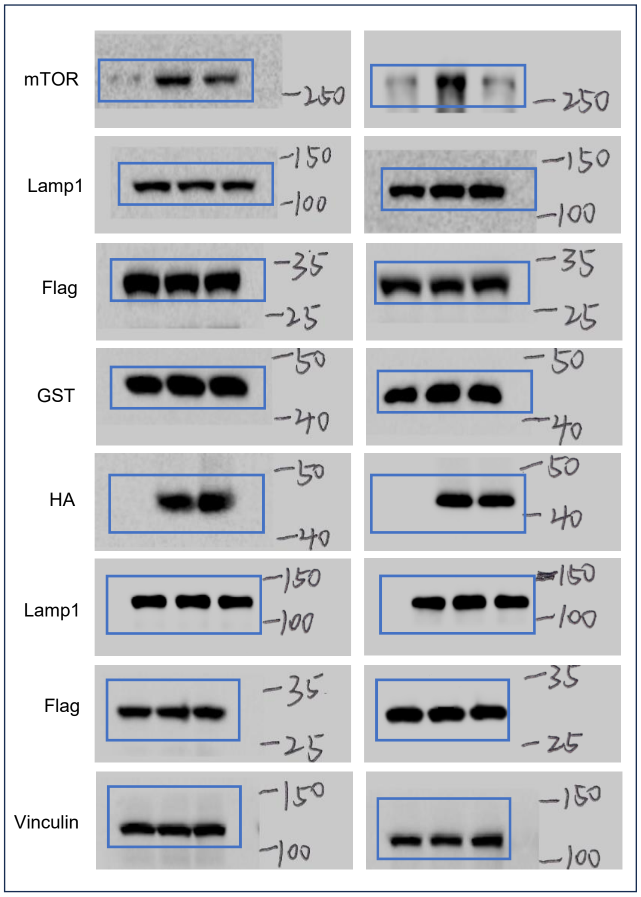

Fig. 5A

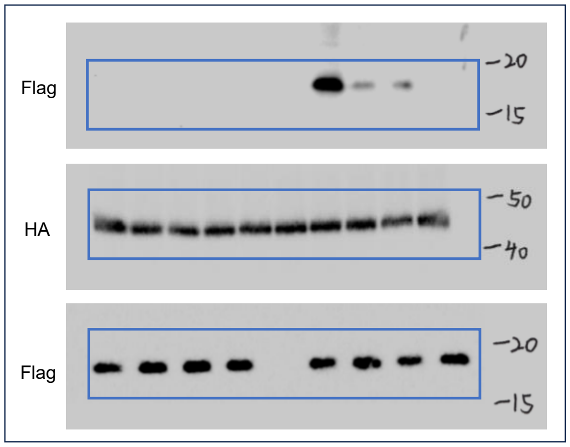

Fig. 5B

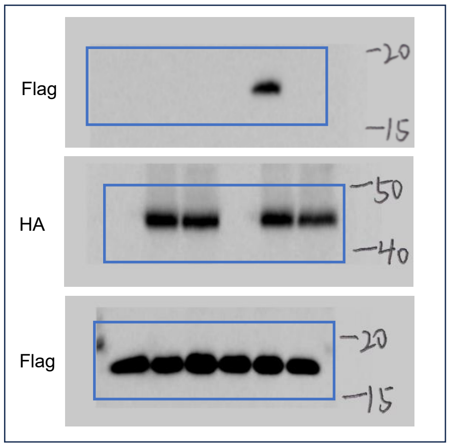

Fig. 5C

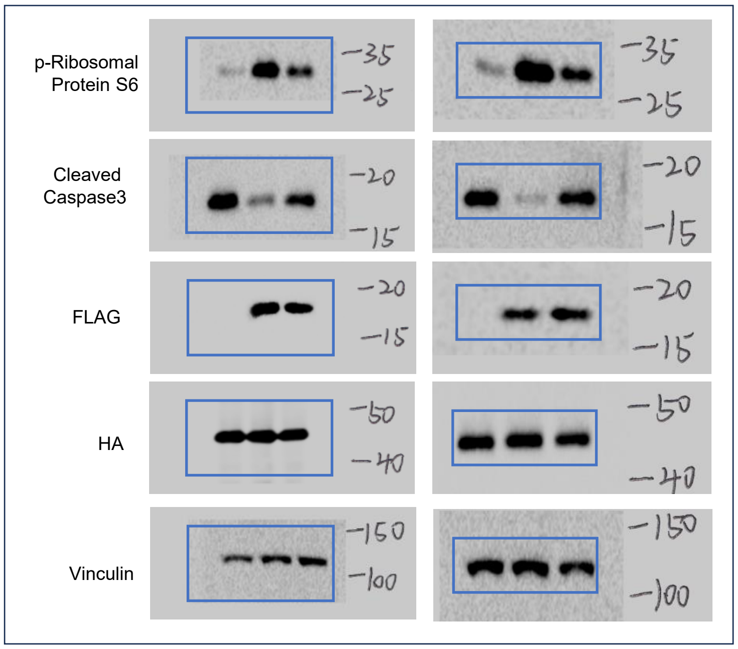

Fig. 5D

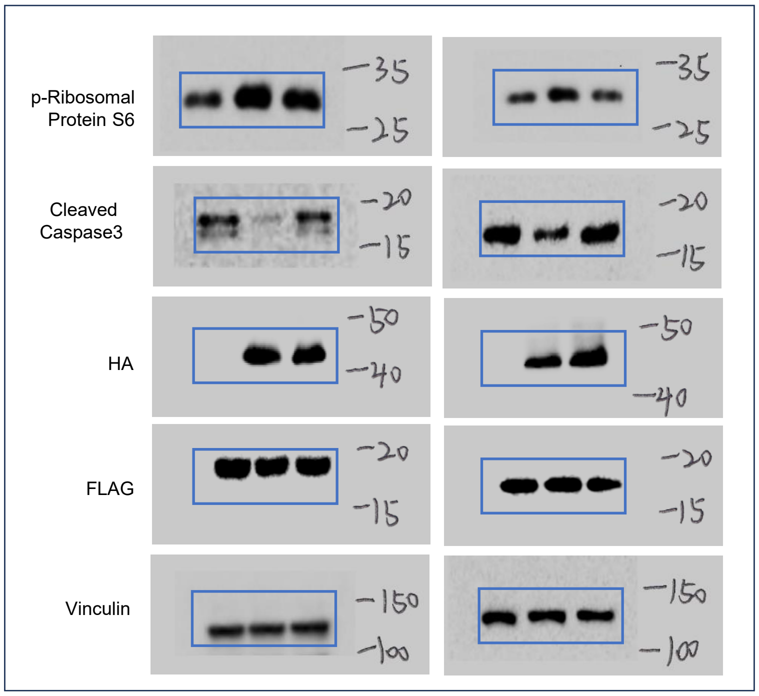

Fig. 5E

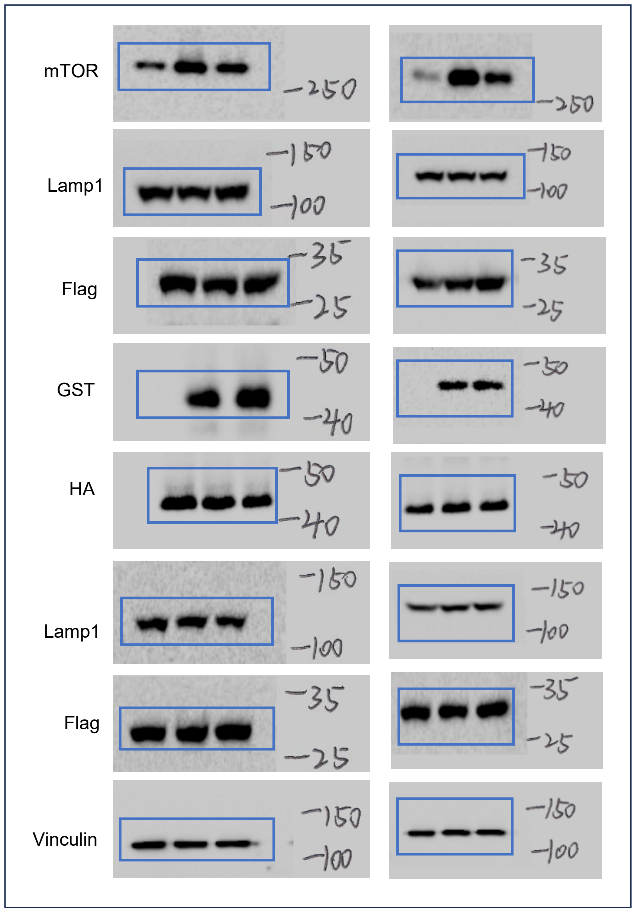

Fig. 5F

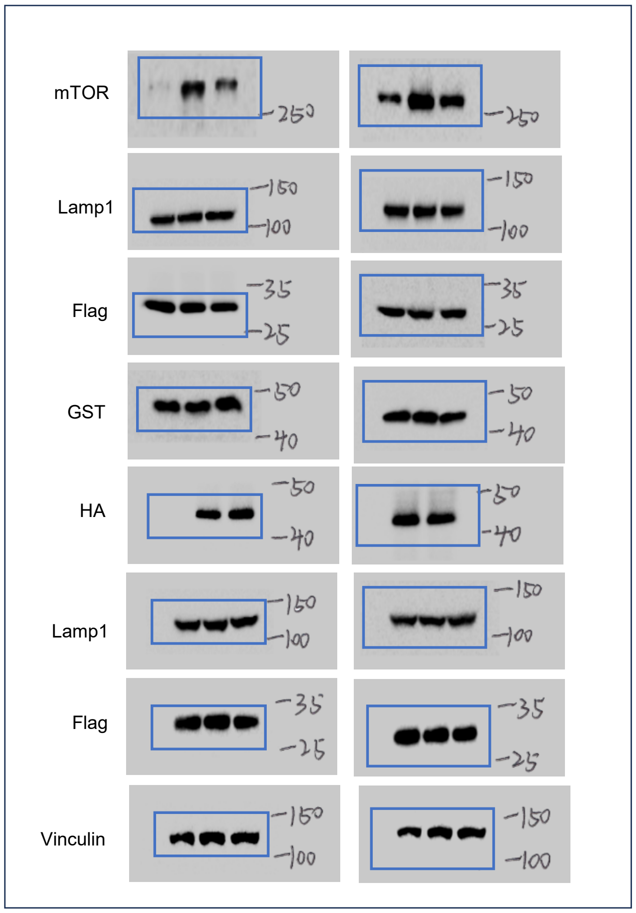

Fig. 6N

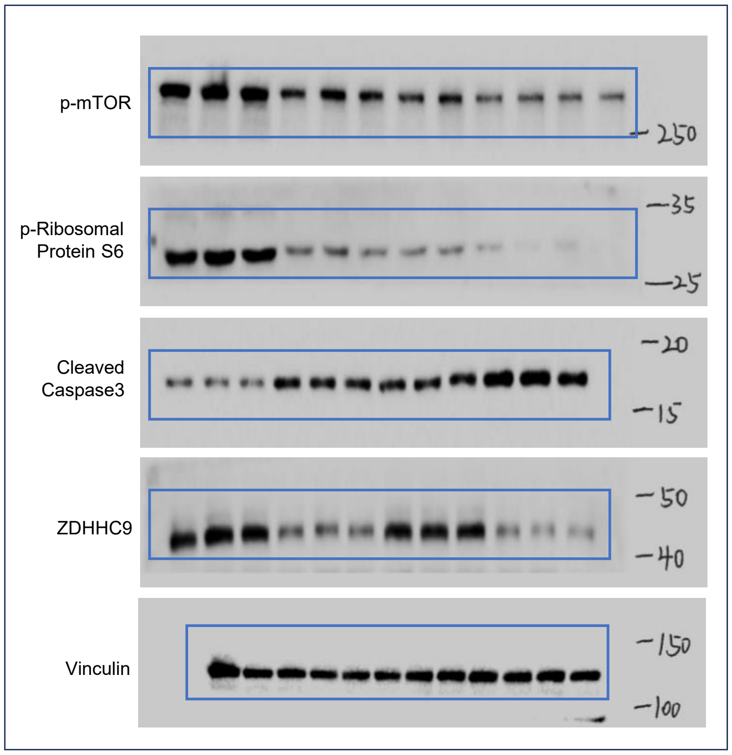

Supplement: Supplementary file 2 — Merge Western blot [file 41419_2026_8558_MOESM2_ESM.pdf]
